# QTL‐seq identifies *BnaFT.A02* and *BnaFLC.A02* as candidates for variation in vernalization requirement and response in winter oilseed rape (*Brassica napus*)

**DOI:** 10.1111/pbi.13421

**Published:** 2020-06-23

**Authors:** Eleri H. Tudor, D. Marc Jones, Zhesi He, Ian Bancroft, Martin Trick, Rachel Wells, Judith A. Irwin, Caroline Dean

**Affiliations:** ^1^ John Innes Centre Norwich Research Park Norwich UK; ^2^ Department of Biology University of York York UK

**Keywords:** flowering time, vernalization, *Brassica napus*, oilseed rape, *FLC*, *FT*

## Abstract

Winter, spring and biennial varieties of *Brassica napus* that vary in vernalization requirement are grown for vegetable and oil production. Here, we show that the obligate or facultative nature of the vernalization requirement in European winter oilseed rape is determined by allelic variation at a 10 Mbp region on chromosome A02. This region includes orthologues of the key floral regulators *FLOWERING LOCUS C* (*BnaFLC.A02*) and *FLOWERING LOCUS T* (*BnaFT.A02*). Polymorphism at *BnaFLC.A02* and *BnaFT.A02*, mostly in cis‐regulatory regions, results in distinct gene expression dynamics in response to vernalization treatment. Our data suggest allelic variation at *BnaFT.A02* is associated with flowering time in the absence of vernalization, while variation at *BnaFLC.A02* is associated with flowering time under vernalizing conditions. We hypothesize selection for *BnaFLC.A02* and *BnaFT.A02* gene expression variation has facilitated the generation of European winter oilseed rape varieties that are adapted to different winter climates. This knowledge will allow for the selection of alleles of flowering time regulators that alter the vernalization requirement of oilseed rape, informing the generation of new varieties with adapted flowering times and improved yields.

## Introduction

Vernalization leads to an acceleration of flowering in response to cold temperatures. Manipulation of the requirement for, and responsiveness to, vernalization has facilitated the generation of novel crop varieties that are adapted to local environments (Jung and Muller, 2009). In seed crops, such as oilseed rape (*Brassica napus*, 2*n* = 4*x* = 38, AACC), predictable and synchronized flowering is essential for a harvestable product.

The close evolutionary relationship between *B. napus* and the reference plant *Arabidopsis thaliana* makes it possible to identify orthologues of flowering time genes (Bancroft *et al*., 2011; Chalhoub *et al*., 2014; Schiessl *et al*., 2017a; Schiessl *et al*., 2014). In *A. thaliana*, allelic variation at two loci, *FRIGIDA* (*AtFRI*) and *FLOWERING LOCUS C* (*AtFLC*), often underlies variation in reproductive strategy (Shindo *et al*., 2005; Stinchcombe *et al*., 2004). Mutations at *AtFRI* that disrupt protein function result in a loss of vernalization requirement (Johanson *et al*., 2000; Michaels *et al*., 2004; Shindo *et al*., 2005), while allelic variation at *AtFLC* is important for fine‐tuning the vernalization response (Ågren *et al*., 2013; Coustham *et al*., 2012; Duncan *et al*., 2015; Grillo *et al*., 2013; Li *et al*., 2014; Li *et al*., 2015; Shindo *et al*., 2006; Strange *et al*., 2011).

Analysis of variation for flowering time in *B. napus* has identified quantitative trait loci (QTL) containing orthologues of *AtFRI*, *AtFLC* and *FLOWERING LOCUS T* (*AtFT*) the floral integrator gene (Chen *et al*., 2018; Ferreira *et al*., 1995; Hou *et al*., 2012; Long *et al*., 2007; Mei *et al*., 2009; Murphy and Scarth, 1994; Nelson *et al*., 2014; Raman *et al*., 2016; Raman *et al*., 2013; Schiessl *et al*., 2017a; Schiessl *et al*., 2015; Schiessl *et al*., 2019; Schiessl *et al*., 2014; Tadege *et al*., 2001; Wang *et al*., 2009; Wang *et al*., 2011; Wu *et al*., 2019; Xu *et al*., 2016; Yi *et al*., 2018). In particular, the *AtFLC* homologue on chromosomes A10 and A02 and the *AtFRI* homologue on chromosome A03 have been identified as major candidates for variation in flowering time in both QTL and genome‐wide association studies.

Despite high sequence conservation between *A. thaliana* and *B. napus*, the presence of multiple orthologous genes complicates translation of the floral regulatory network from reference diploid to polyploid crop species (Jones *et al*., 2018). Multiple orthologues of flowering time genes have been preferentially retained and are expressed in *B. napus* (Jones *et al*., 2018; Schiessl *et al*., 2017a; Schiessl *et al*., 2014); however, many studies have predominately focused on Chinese semi‐winter and Australian or Canadian spring varieties of oilseed rape (Hou *et al*., 2012; Long *et al*., 2007; Raman *et al*., 2016; Raman *et al*., 2013; Wang *et al*., 2009; Wang *et al*., 2011; Xu *et al*., 2016). Here, we investigate the genetic variation underlying flowering time differences between European winter oilseed rape varieties. An F_2_ population was generated from a biparental cross between an early flowering European winter oilseed rape variety Cabriolet and a late flowering European winter oilseed rape variety Darmor. A QTL‐seq approach (Takagi *et al*., 2013), which combines bulked segregant analysis (Giovannoni *et al*., 1991; Michelmore *et al*., 1991) with whole‐genome resequencing (Das *et al*., 2015; Illa‐Berenguer *et al*., 2015; Lu *et al*., 2014; Singh *et al*., 2016; Takagi *et al*., 2013; Wang *et al*., 2016), was used to identify QTL for flowering time with and without vernalization. A major QTL was identified on chromosome A02 which includes orthologues of *AtFLC* (*BnaFLC.A02*) and *AtFT* (*BnaFT.A02*). Allelic variation at *BnaFLC.A02* accounted for a higher proportion of variation in flowering time following a six‐week vernalization treatment, while polymorphism at *BnaFT.A02* co‐segregated with flowering time in the absence of vernalization. Cis‐polymorphism and altered gene expression dynamics were detected at both genes, revealing parallels with natural accessions of *A. thaliana*.

## Results

### European winter oilseed rape segregates for an obligate or facultative vernalization requirement

**Figure 1:**
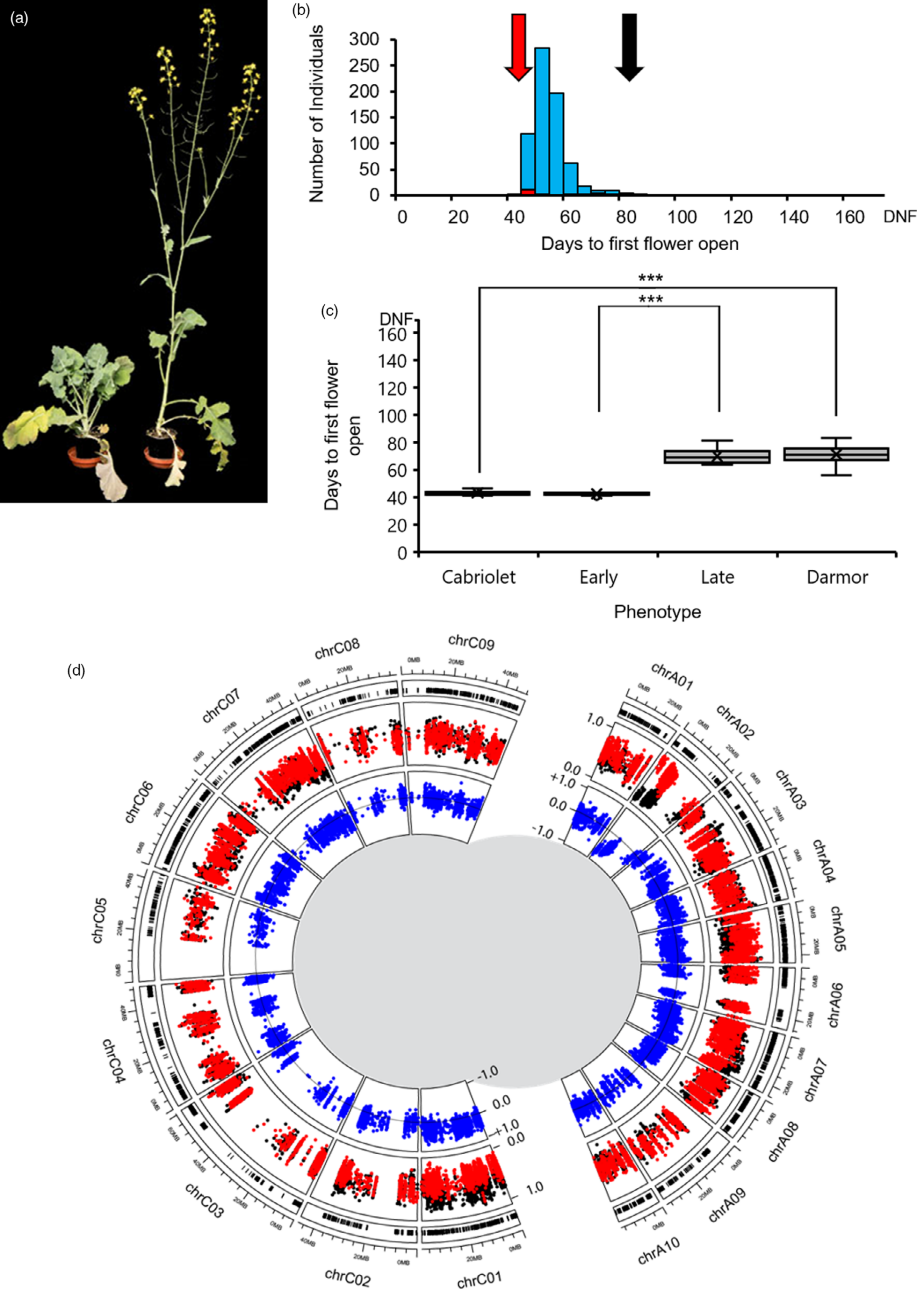
Variation for vernalization response is present in European winter oilseed rape. (a) Flowering phenotype of the late flowering variety Darmor (left) and early flowering variety Cabriolet (right) at 50 days’ growth after a 6‐week vernalization treatment. (b) Frequency distribution of flowering time of Cabriolet, Darmor and 704 F_2_ lines under VERN treatment. Flowering time was recorded as days to first flower open from the first day plants were transferred to the poly‐tunnel. (c) Flowering time distribution of Cabriolet, the early flowering bulk, the late flowering bulk and Darmor under VERN treatment; box and whisker plots represent the mean and quartile values. Flowering time was recorded as days to first flower open from the first day plants were transferred to the poly‐tunnel. (d) Results of a QTL‐seq approach for mapping flowering time in European winter oilseed rape under VERN treatment. Outer circle: the distribution of Cabriolet variants detected in the bulks plotted against the chromosomal position according to Darmor‐*bzh*. Middle circle: the SNP index values calculated for each variant in the early bulk (red) and the late bulk (black) plotted in genome order according to Darmor‐*bzh*. Inner circle: the difference between SNP index values between the bulks plotted as the ΔSNP index (blue) against the chromosomal position according to Darmor‐*bzh*, a ΔSNP index equal to zero representing no deviation in allele segregation between the bulks is plotted as a horizontal line. Each genome is plotted as separate half circles.

To investigate variation in flowering time within European winter oilseed rape with and without vernalization, the winter oilseed rape varieties Cabriolet and Darmor (Figure 1a) were used as parents to construct a segregating F_2_ population. Four‐week‐old plants from this population were either given a six‐week vernalization treatment at 5 °C (VERN) before being transferred to an unheated poly‐tunnel or grown on under ambient temperature conditions (no vernalization, NVERN) in the same poly‐tunnel during the spring and summer of 2017 when the daylength always exceeded 12 h. Temperature and relative humidity were recorded for the duration of the experiment (Figure S1), and plants experienced an average temperature and relative humidity of 18.96 °C and 68.24%, respectively. Flowering time was recorded from the date of transfer to the poly‐tunnel and excluded the number of days growth before transfer.

**Figure 2:**
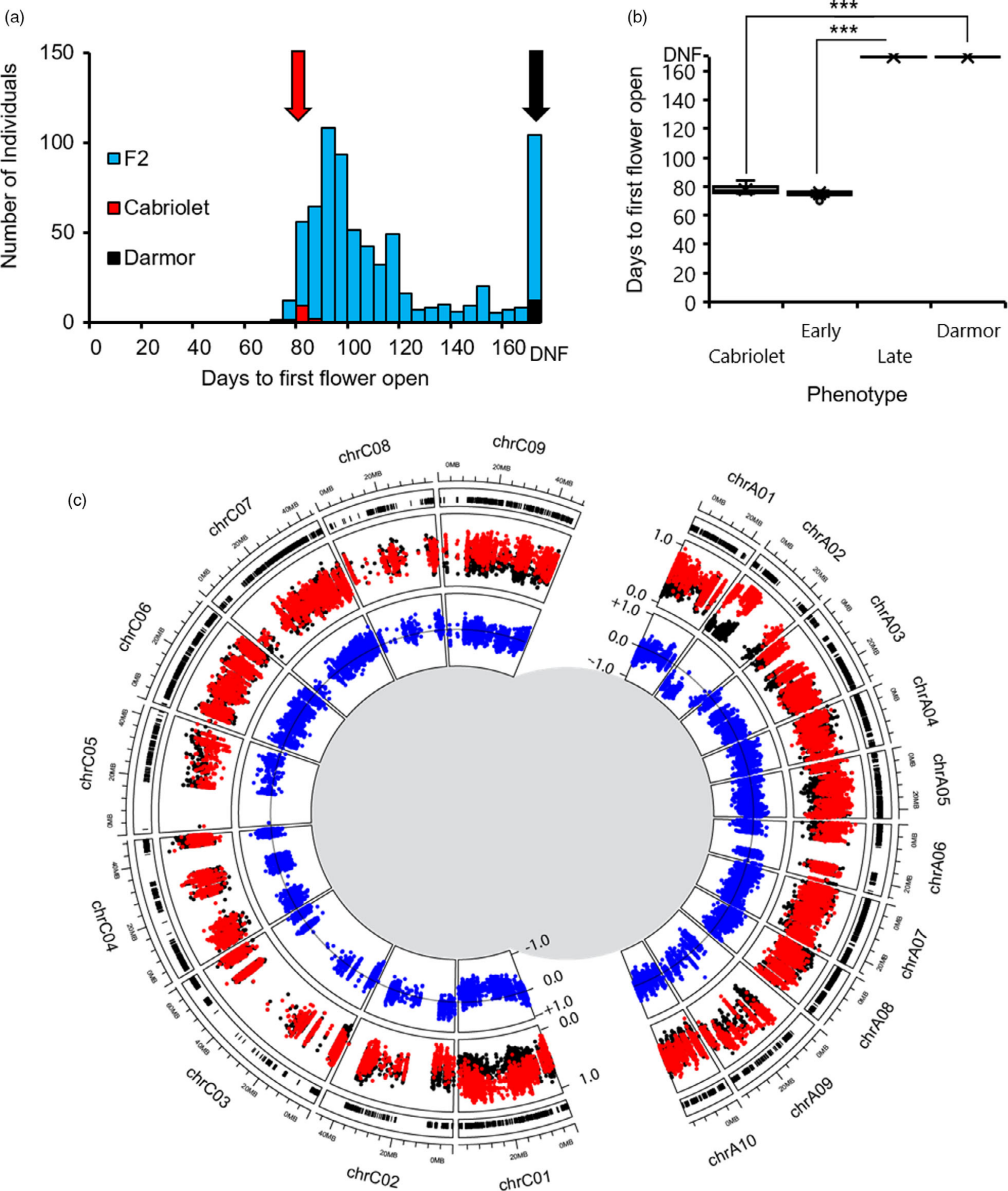
Variation for vernalization requirement is present in European winter oilseed rape. (a) Frequency distribution of flowering time of Cabriolet, Darmor and 708 F_2_ lines under NVERN treatment. Flowering time was recorded as days to first flower open from the first day plants were transferred to the poly‐tunnel. (b) Flowering time distribution of Cabriolet, the early flowering bulk, the late flowering bulk and Darmor under NVERN treatment; box and whisker plots represent the mean and quartile values. Flowering time was recorded as days to first flower open from the first day plants were transferred to the poly‐tunnel. (c) Results of a QTL‐seq approach for mapping flowering time in European winter oilseed rape under NVERN treatment. Outer circle: the distribution of Cabriolet variants detected in the bulks plotted against the chromosomal position according to Darmor‐*bzh*. Middle circle: the SNP index values calculated for each variant in the early bulk (red) and the late bulk (black) plotted in genome order according to Darmor‐*bzh*. Inner circle: the difference between SNP index values between the bulks plotted as the ΔSNP index (blue) against the chromosomal position according to Darmor‐*bzh*, a ΔSNP index equal to zero representing no deviation in allele segregation between the bulks is plotted as a horizontal line. Each genome is plotted as separate half circles.

Under both treatments, Darmor flowered significantly later than Cabriolet (*P* < 0.001, Mann–Whitney *U*‐test, Figure 1a,c and Figure 2a,b). Under VERN, Cabriolet flowered within an average of 42.92 days after transfer to the poly‐tunnel (which equated to 114.92 days after sowing), while Darmor flowered within an average of 71.25 days (141.25 days after sowing) and exhibited a broader range of flowering times, indicative of incomplete vernalization (Table S1). Under NVERN, Cabriolet flowered within an average of 78 days (106 days after sowing) and therefore exhibited a facultative vernalization requirement, while Darmor did not flower within the timeframe of the experiment and exhibited an obligate requirement.

Seven hundred and four F_2_ lines were assessed for flowering time following the VERN treatment. Flowering time under these conditions exhibited a continuous distribution, with a right‐hand skew suggesting flowering time in *B. napus* is quantitatively inherited (Figure 1b). In parallel, 708 F_2_ lines were assessed for flowering time under the NVERN treatment. Flowering time NVERN exhibited a bimodal distribution including a subset of 86 lines that did not flower (Figure 2a). The earliest lines flowered 70 days after transfer to the poly‐tunnel. Under both treatments, no significant difference was detected between F_2_ lines from each reciprocal cross (NVERN *P* = 0.426 ANOVA, VERN *P* = 0.219 ANOVA) and therefore no maternal effect on flowering time was detected. Very little transgressive segregation was observed, suggesting the two parent varieties were representative of the extremes of flowering time variation in this genetic background.

### A single genomic region on chromosome A02 is associated with flowering time with and without vernalization

To identify the major genomic regions important for flowering time in the F_2_ population, lines were selected for bulking based on flowering time phenotype. Four DNA bulks were generated, two bulks for each treatment, and included approximately 5% of the population representing the earliest and latest flowering lines (Figures 1c and 2b). Genomic DNA from the parent plants (Cabriolet and Darmor) and the four bulks were sequenced, generating 705 million clean sequencing reads with a Q30 score of at least 90.2% (Table S1).

To identify high confidence (read depth> 20, >95% base call) variants (SNPs and small InDels (<9bp)) between Cabriolet and Darmor, we compared the sequencing reads from Darmor to the Darmor‐*bzh* reference genome (Chalhoub *et al*., 2014). Darmor‐*bzh* was generated by introgressing the dwarf *BREIZH* (*Bzh*) locus from a line called B192 into Darmor and is therefore predicted to carry genetic differences (Foisset *et al*., 1995). Twelve thousand five hundred and twenty two high confidence variants were identified in Darmor compared with Darmor‐*bzh,* and 9561 of these could be anchored to a chromosomal position (Figure S2). The density and location of SNPs suggest large genomic blocks vary between Darmor and Darmor‐*bzh* and this variation was accounted for when comparing Cabriolet and Darmor.

Thirty nine thousand seven hundred and seventeen high confidence variants were identified in Cabriolet compared with Darmor, and 32 773 could be anchored to a chromosomal position (Figure S3). SNP and ΔSNP indices were calculated for each variant under both VERN and NVERN treatments and plotted against their genomic position according to the Darmor‐*bzh* reference sequence (Chalhoub *et al*., 2014) in Figures 1d and 2c, respectively.

**Figure 3:**
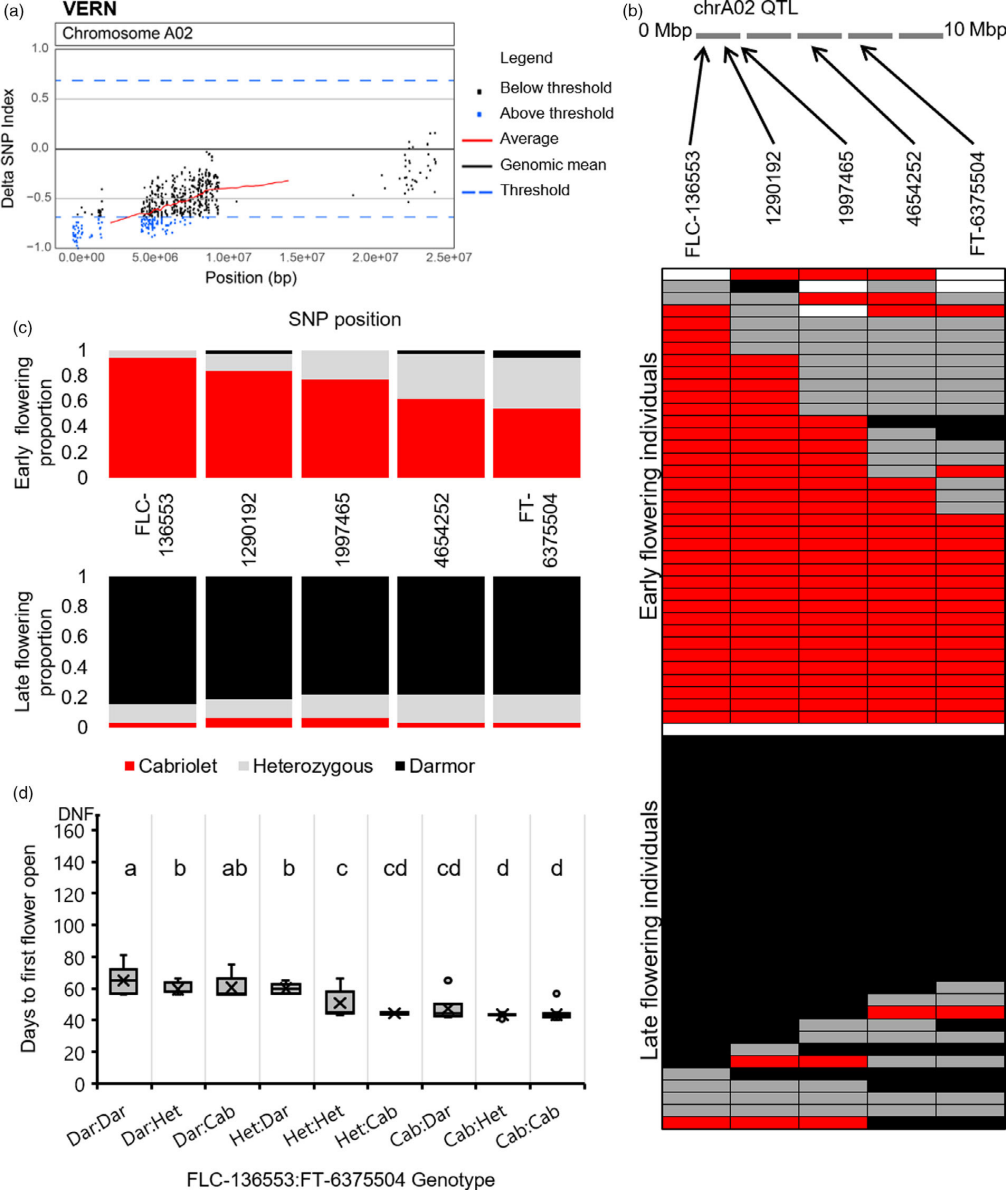
A VERN QTL for flowering time is located on chromosome A02. (a) ΔSNP index plot of chromosome A02 under VERN treatment. ΔSNP index values are plotted in Brassica chromosome A02 order, and values found within the top 1% of ΔSNP index values are coloured blue. (b) Validation of the VERN QTL region on chromosome A02 by KASP assay. Upper panel: schematic of the QTL region on chromosome A02 with the relative locations of SNPs targeted by KASP assay are highlighted. Lower panel: the genotype of all F_2_ lines within the VERN DNA bulks screened at five SNP positions within the QTL; SNPs homozygous for the Cabriolet allele are coloured red, SNPs homozygous for the Darmor allele are coloured black, SNPs that are heterozygous are coloured grey, and SNP genotypes that could be not determined are left white. The grid is divided into early flowering bulk and the late flowering bulk. (c) The proportion of F_2_ lines that were homozygous for Cabriolet alleles (red), homozygous for Darmor alleles (black) or heterozygous (grey) at five SNP positions on chromosome A02 in the DNA bulks under VERN treatment. (d) The flowering time phenotype under VERN treatment of F_2_ lines genotyped for SNP markers FLC‐136553 and FT‐6375504 (Cab = Cabriolet, Dar = Darmor, Het = heterozygous). Letters above the columns indicate significant differences determined by multiple pairwise comparisons using Mann–Whitney U‐test‐with an α‐value of 0.05. Flowering time was recorded as days to first flower open from the first day plants were transferred to the poly‐tunnel.

**Figure 4:**
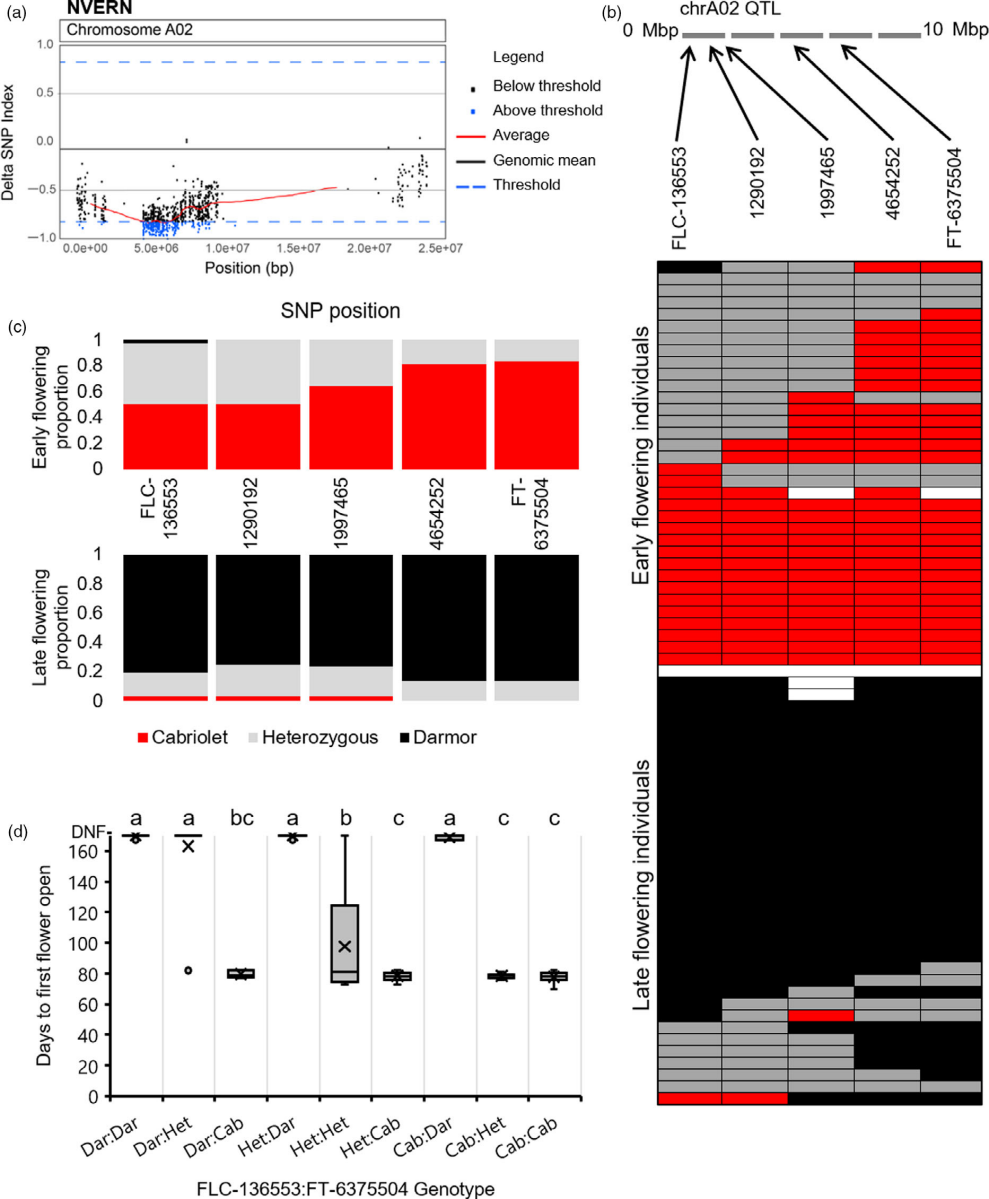
A NVERN QTL for flowering time is located on chromosome A02. (a) ΔSNP index plot of chromosome A02 under NVERN treatment. ΔSNP index values are plotted in Brassica chromosome A02 order, and values found within the top 1% of ΔSNP index values are coloured blue. (b) Validation of the NVERN QTL region on chromosome A02 by KASP assay. Upper panel: schematic of the QTL region on chromosome A02 with the relative locations of SNPs targeted by KASP assay are highlighted. Lower panel: the genotype of all F_2_ lines within the NVERN DNA bulks screened at five SNP positions within the QTL; SNPs homozygous for the Cabriolet allele are coloured red, SNPs homozygous for the Darmor allele are coloured black, SNPs that are heterozygous are coloured grey, and SNP genotypes that could be not determined are left white. The grid is divided into the early flowering bulk and the late flowering bulk. (c) The proportion of F_2_ lines that were homozygous for Cabriolet alleles (red), homozygous for Darmor alleles (black) or heterozygous (grey) at five SNP positions on chromosome A02 in the DNA bulks under NVERN treatment. (d) The flowering time phenotype under NVERN treatment of F_2_ lines genotyped for SNP markers FLC‐136553 and FT‐6375504 (Cab = Cabriolet, Dar = Darmor, Het = heterozygous). Letters above the columns indicate significant differences determined by multiple pairwise comparisons using Mann–Whitney U‐test‐with an α‐value of 0.05. Flowering time was recorded as days to first flower open from the first day plants were transferred to the poly‐tunnel.

To identify genomic regions responsible for the difference in flowering time between the early and late flowering bulks, we chose absolute ΔSNP index threshold values of 0.686 and 0.828 for VERN and NVERN treatments, respectively. These threshold values represented the top 1% of absolute ΔSNP index values across the whole genome for each treatment. For both VERN and NVERN treatments, a deviation in SNP index values was detected across a single 10Mbp region on chromosome A02 and contained numerous absolute ΔSNP index values above these thresholds (Figures 1d, 2c, 3a and 4a). The direction of ΔSNP index values towards −1.0 in both VERN and NVERN treatments suggests the genetic contribution for early flowering was determined by Cabriolet and late flowering by Darmor. By calculating an average ΔSNP index value for every 100‐variant window across the chromosome, we could determine a peak of association within the QTL region. For VERN, a peak in average ΔSNP index values was detected 0–2.5 Mbp from the upper arm of chromosome A02 (Figure 3a). However, for NVERN this peak was detected at a region 5–7.5 Mbp from the upper arm of chromosome A02 (Figure 4a). This suggested that different genes within the same 10 Mbp region on chromosome A02 were responsible for the differences in flowering time between the bulks under VERN and NVERN treatments.

There are 1937 genes annotated within the 10Mbp QTL region on chromosome A02 according to the Darmor‐*bzh* reference genome. Based on homology with genes in *A. thaliana*, 21 are hypothesized to contribute to the control of flowering time and only six of these genes are polymorphic between Cabriolet and Darmor (Table 1). Likely candidates that associate with variation in flowering time between Cabriolet and Darmor include *BnaA02g00370d* (hereafter referred to as *BnaFLC.A02*), a homologue of *AtFLC* and a MADS‐box transcription factor responsible for the inhibition of flowering until after vernalization, and *BnaA02g12130D* (hereafter referred to as *BnaFT.A02*) a homologue of *AtFT*, a gene considered an integrator of photoperiod and vernalization signals, and a promoter of flowering. The other four potential candidates were homologues of *Gibberellin 20 oxidase 2*, *EMBRYONIC FLOWER 2*, *CSTF64* and *TBP2*. Interestingly, no deviation in either SNP or ΔSNP indices was found on chromosomes A03, A10, C02, C03 and C09 where other orthologues of *AtFLC* and orthologues of *AtFRI* have been mapped. No high confidence variants were detected at the major candidate genes for flowering time, *BnaFLC.A10* and *BnaFRI.A03* (Hou *et al*., 2012; Wang *et al*., 2011) (Figure S4). Both Cabriolet and Darmor carry the previously described winter‐type allele of both *BnaFLC.A10* (Hou *et al*., 2012; Song *et al*., 2020) and *BnaFRI.A03* (Wang *et al*., 2011) which suggests neither gene contributes to flowering time variation within this population.

**Table 1:** Candidate flowering time genes found within the QTL region for vernalisation requirement and response on chromosome A02

Position (bp)	Gene name	Genetic status in Cabriolet	Homologue in *A. thaliana*	Function in *A. thaliana*
134,159 – 138,121	BnaA02g00370d	Polymorphic	AT5G10140, *FLOWERING LOCUS C, FLC*	MADS‐box transcription factor, repressor of flowering, responsive to vernalization
408,979 – 413,943	BnaA02g01030D	Conserved	AT5G11530, *EMBRYONIC FLOWER 1, EMF1*	Involved in reproductive development
565,722 – 567,762	BnaA02g01270D	Conserved	AT5G12840, Nuclear transcription factor Y subunit A‐1, *NFYA1*	Expressed in reproductive tissue
773,658 – 778,149	BnaA02g01670D	Conserved	AT5G13480, *FY*	Involved in regulation of flowering time, affects *FCA* mRNA processing
893,325 – 894,144	BnaA02g01960D	Conserved	AT5G14010, *KNUCKLES, KNU*	Transcription factor, mediates repression of *WUS* in floral meristem determinacy control
2,115,552 – 2,118,359	BnaA02g04770D	Conserved	AT5G20240, *PISTILLATA, PI*	Floral homeotic gene, MADS domain transcription factor, required for specification of petal and stamen identities
3,037,543 – 3,043,336	BnaA02g06350D	Conserved	AT5G60410, *ATSIZ1*	
3,106,207 – 3,108,826	BnaA02g06490D	Conserved	AT5G60120, Target of early activation tagged (EAT)2, *TOE2*	
3,111,280 – 3,113,426	N/A	Conserved	AT5G60100, *Pseudo‐response regulator 3*, *PRR3*	*PRR3* transcript levels vary in circadian pattern
3,320,312 – 3,321,741	BnaA02g07010D	Conserved	AT5G59560, *SENSITIVITY TO RED LIGHT REDUCED 1*, *SRR1*	Required for normal oscillator function during circadian rhythm
3,685,147 – 3,687,730	BnaA02g07770D	Conserved	AT5G58230, *MSI1*	Required for the transition to flowering
3,861,836 – 3,864,833	BnaA02g08140D	Conserved	AT5G57380, *VERNALIZATION INSENSITIVE 1,* *VIN3*	Plant homeodomain protein, part of polycomb group complex of proteins, has a role in establishing *FLC* repression during vernalization
5,851,901 – 5,853,606	BnaA02g11210D	Polymorphic	AT5G51810, *Gibberellin 20 oxidase 2*, *GA20OX2*	
5,948,452 – 5,953,119	BnaA02g11340D	Polymorphic	AT5G51230, *EMBRYONIC FLOWER 2, EMF2*	Polycomb group protein, a negative regulator of reproductive development
6,375,937 – 6,378,901	BnaA02g12130D	Polymorphic	AT1G65480, *FLOWERING LOCUS T, FT*	A promoter of flowering, expressed in leaves and is induced by long day treatment
7,870,038 – 7,871,424	BnaA02g14040D	Conserved	AT1G68840, *RAV2*	
8,998,899 – 9,001,958	BnaA02g15530D	Polymorphic	AT1G71800, *CSTF64*	RNA 3’‐end‐processing factor of antisense *FLC* transcript, mediates silencing of *FLC* gene
9,423,231 – 9,429,930	BnaA02g15970D	Polymorphic	AT1G72390, *TBP2*	
9,980,484 – 9,983,268	BnaA02g16710D	Conserved	AT2G18915, *Adagio protein 2*, *ADO2*, *LKP2*	
10,268,028 – 10,270,134	BnaA02g17110D	Conserved	AT1G75060, *BLH3*	
10,804,098 – 10,805,192	N/A	Conserved	AT4G20370, *TWIN SISTER OF FT, TSF*	A promoter of flowering and a homologue of *FT*, *FT* and *TSF* play overlapping roles in the transition to flowering

Flowering time gene position on chromosome A02, the gene name according to the Darmor‐*bzh* genome, and genetic status in Cabriolet compared with Darmor are listed. The homologous gene name and function in *A. thaliana* is included for reference.

### The QTL is not the result of a homeologous exchange between the A02 and C02 chromosomes

The comparatively recent origin of amphidiploid *B. napus* has been shown to result in frequent exchanges between homeologous regions of the A and C diploid genomes. One region where such exchanges are reported to occur is at the top of chromosomes A02 and C02 (Chalhoub *et al*., 2014, He *et al*., 2017). To determine whether a homeologous exchange (HE) contributed to the QTL on A02 in this population, we conducted HE analysis on the parental Darmor and Cabriolet genome sequences as per He *et al*. (2017). Some differences were observed (Figure S5), including a HE in Darmor compared with Cabriolet on chromosome C02. Although located within the QTL region, this HE did not overlap with the most strongly associated variants identified by QTL‐seq and is therefore not likely to be the cause of the QTL (Figure S5B).

### Validation of the QTL region by KASP markers reveals different genomic regions of chromosome A02 are associated with flowering time with and without vernalization

To validate the region on chromosome A02 identified by QTL‐seq, KASP (Kompetitive allele‐specific PCR) primers were designed to target a 5Mbp region between the two most promising candidate genes, *BnaFLC.A02* and *BnaFT.A02*, within the QTL. For both NVERN and VERN treatments, five SNPs including a SNP within *BnaFLC.A02* (SNP 136553 hereafter referred to as FLC‐136553) and within the promoter of *BnaFT.A02* (SNP 6375504 hereafter referred to as FT‐6375504) (Table S2) were targeted to determine the segregation of alleles in the earliest and latest flowering F_2_ lines. We first screened the lines that were included in the DNA bulks of the VERN and NVERN QTL‐seq analyses. Each line was scored for segregation of alleles at the five SNP markers, and all five SNPs were assigned a Cabriolet (Cab), Darmor (Dar) or heterozygous (Het) allele. The genotypes of each line included in the DNA bulks are summarized in Figures 3b and 4b.

Using these genotyping results, we were able to determine the frequency at which the alleles appeared within the DNA bulks. Within the VERN DNA bulks, all lines in the early flowering bulk were either homozygous (94.4%) or heterozygous (5.6%) for the Cabriolet allele at SNP marker FLC‐136553 within *BnaFLC.A02* (Figure 3c). In contrast, a majority of lines within the late flowering bulk were either homozygous (84.4%) or heterozygous (12.5%) for the Darmor allele at the same SNP marker (Figure 3c). The proportion of lines in both bulks that were homozygous for each marker decreased across the 5Mbp region downstream towards SNP marker FT‐6375504 at *BnaFT.A02*. At this position, 54.3% of lines within the early flowering bulk were homozygous for the Cabriolet allele, and 78.1% of lines within the late flowering bulk were homozygous for the Darmor allele. We conclude that homozygosity for Darmor alleles at a region that includes *BnaFLC.A02* and *BnaFT.A02* is important for determining lateness of flowering, but homozygosity for Cabriolet alleles at a region that includes only *BnaFLC.A02* is required for determining earliness of flowering. In agreement with the QTL‐seq analysis, a significantly higher proportion of lines were homozygous at *BnaFLC.A02* compared with *BnaFT.A02* (*P* < 0.001, Wilcoxon matched‐pairs test), confirming allelic variation at a region that includes *BnaFLC.A02* is most closely associated with variation in flowering time within the F_2_ population under VERN conditions.

The same genotyping analysis was performed for the lines included in the DNA bulks of the NVERN treatment. Within the DNA bulks, all lines in the early flowering bulk were either homozygous (83.3%) or heterozygous (16.7%) for the Cabriolet allele at SNP marker FT‐6375504, found within the promoter of *BnaFT.A02* (Figure 4c). In contrast, all lines in the late flowering bulk were either homozygous (86.1%) or heterozygous (13.9%) for the Darmor allele at the same SNP marker (Figure 4c). The proportion of lines in both bulks that were homozygous for each marker decreased across the 5Mbp region upstream towards SNP marker FLC‐136553 at *BnaFLC.A02*. At this position, 50% of lines within the early flowering bulk were homozygous for the Cabriolet allele, and 80.6% of lines within the late flowering bulk were homozygous for the Darmor allele. We therefore conclude that homozygosity at a region that includes *BnaFLC.A02* and *BnaFT.A02* is important for determining late flowering due to the high incidence of Darmor alleles within the late flowering bulk. However, homozygosity of the Cabriolet allele of *BnaFLC.A02* is not required to determine early flowering under these conditions. In agreement with the QTL‐seq analysis, a significantly higher proportion of lines were homozygous at *BnaFT.A02* compared with *BnaFLC.A02* (*P* = 0.035, Wilcoxon matched‐pairs test), confirming allelic variation at a region that includes *BnaFT.A02* is most closely associated with variation in flowering time within the F_2_ population under NVERN conditions.

To further validate this, we screened the next 78 lines, 94 lines in total, from each tail of the distribution in flowering time (Figures 1b and 2a) within the F_2_ population under both VERN and NVERN treatments using the same five SNP markers. All nine possible genotypic combinations of SNP markers were detected in the F_2_ population (Figures 3d and 4d). Significant differences in flowering times were observed between the nine genotypes (*P* < 0.001, Mann–Whitney *U*‐test; Figures 3d and 4d) and under both treatments, lines homozygous for FLC‐136553‐Cab and FT‐6375504‐Cab flowered significantly earlier than lines homozygous for FLC‐136553‐Dar and FT‐6375504‐Dar. Lines heterozygous for both markers exhibited an intermediate flowering time, while the flowering time of recombinant lines with one homozygous and one heterozygous allele was dependent on the genotype of the homozygous allele.

Within the DNA bulks, the instance of homozygous recombinant lines carrying *BnaFLC.A02* from one parent and *BnaFT.A02* from the other parent was rare (Figures 3b and 4b). This suggests both genes are important for the extreme early and late flowering phenotypes within the population. By expanding our screen to 94 lines from each tail of the distribution, we identified 11 and 14 recombinant lines under VERN and NVERN, respectively. The flowering time of recombinant lines homozygous for SNP marker FLC‐136553 from one parent and homozygous for SNP marker FT‐6375504 from the other parent (FLC‐136553‐Cab and FT‐6375504‐Dar, or FLC‐136553‐Dar and FT‐6375504‐Cab) was dependent on vernalization treatment, although not always flowering as early or late as their non‐recombinant counterparts. Under VERN treatment, lines with genotypic combination FLC‐136553‐Cab and FT‐6375504‐Dar flowered significantly earlier than lines with genotypic combination FLC‐136553‐Dar and FT‐63755040‐Cab (Figure 3d). In contrast, under NVERN treatment, lines with a genotypic combination FLC‐136553‐Cab and FT‐6375504‐Dar flowered significantly later than lines with combination FLC‐136553‐Dar and FT‐6375504‐Cab (Figure 4d). This result again supports the hypothesis that allelic variation at *BnaFLC.A02* controls flowering time under VERN treatment, while allelic variation at *BnaFT.A02* is responsible for differences in flowering time under NVERN treatment. A single outlier line with genotypic combination FLC‐136553‐Cab and FT‐6375504‐Dar that flowered late under VERN treatment reveals that other minor effect genes may also contribute to variation in flowering time within the population.

### Cis‐polymorphism at *BnaFLC.A02* affects the stability of *FLC* silencing

**Figure 5:**
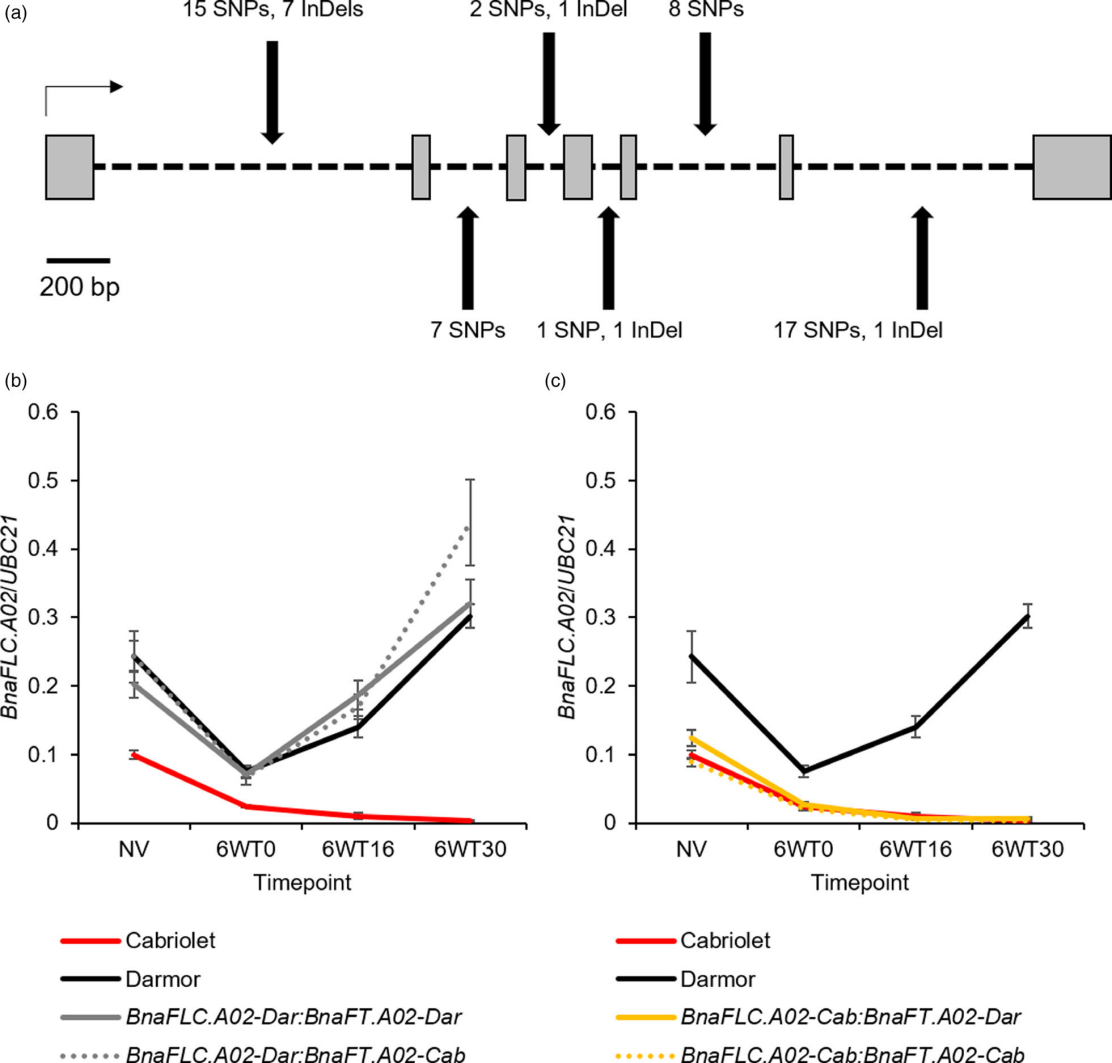
DNA sequence and gene expression variation of *BnaFLC.A02* in Cabriolet and Darmor. (a) The polymorphisms identified at *BnaFLC.A02* in Cabriolet compared with Darmor are highlighted with black arrows, grey boxes represent exons, black dashed lines represent introns, and the black horizontal arrow represents the direction of transcription. (b) Normalized expression of *BnaFLC.A02* in Cabriolet, Darmor and genotyped F_2_ lines for *BnaFLC.A02* and *BnaFT.A02*, as measured by quantitative RT‐PCR before (NV) and after a six‐week vernalization treatment (6WT0, 6WT16, 6WT32), error bars denote one standard error around the mean calculated from at least three biological replicates. (c) Normalized expression of *BnaFLC.A02* in Cabriolet, Darmor and genotyped F_2_ lines for *BnaFLC.A02* and *BnaFT.A02*, as measured by quantitative RT‐PCR before (NV) and after a six‐week vernalization treatment (6WT0, 6WT16, 6WT32), error bars denote one standard error around the mean calculated from at least three biological replicates.

*BnaFLC.A02* was previously cloned by Tadege *et al*. (2001) and Zou *et al*. (2012) and, like *AtFLC*, it is organized into seven exons and six introns. *BnaFLC.A02* was amplified and sequenced from the two parent lines. Compared with Darmor *BnaFLC.A02* (hereafter referred to as *BnaFLC.A02‐Dar*), the Cabriolet allele (hereafter referred to as *BnaFLC.A02‐Cab*) had 50 SNPs and 10 InDels; all were located within introns (Figure 5a).

The presence of non‐coding sequence variation at *BnaFLC.A02* prompted us to investigate the expression of the two *BnaFLC.A02* alleles by quantitative RT‐PCR before (NV) and after six‐week vernalization treatment in Cabriolet, Darmor and in a panel of F_2_ lines that had been genotyped for their alleles at *BnaFLC.A02* and *BnaFT.A02*. *BnaFLC.A02* expression was consistently lower in leaves of Cabriolet than Darmor plants at 28 days after sowing (Figure 5b,c and Figure S6). The two *BnaFLC.A02* alleles also exhibited differences in expression dynamics following a six‐week vernalization treatment at 5 °C. Both alleles were repressed by six‐week vernalization, but *BnaFLC.A02‐Cab* was repressed to lower levels compared to *BnaFLC.A02‐Dar*. On return to warm conditions, *BnaFLC.A02‐Cab* expression remained low and therefore its expression was stably repressed, while *BnaFLC.A02‐Dar* reactivated to expression levels not dissimilar to those levels detected before vernalization. The F_2_ lines could be grouped into four genotypes dependent on their homozygous alleles at *BnaFLC.A02* and *BnaFT.A02* (Cab:Cab, Dar:Dar, Cab:Dar, Dar:Cab). F_2_ lines that were homozygous for *BnaFLC.A02‐Cab* exhibited similar expression patterns to those detected in Cabriolet, and lines carrying *BnaFLC.A02‐Dar* showed expression dynamics similar to Darmor plants at 28 days. *BnaFLC.A02* expression in recombinant F_2_ lines (*BnaFLC.A02‐Cab* and *BnaFT.A02‐Dar*, or *BnaFLC.A02‐Dar* and *BnaFLC.A02‐Cab*) was independent of *BnaFT.A02* (Figure 5b,c and Figure S6).

### *BnaFT.A02* is highly expressed in Cabriolet but not in Darmor

**Figure 6:**
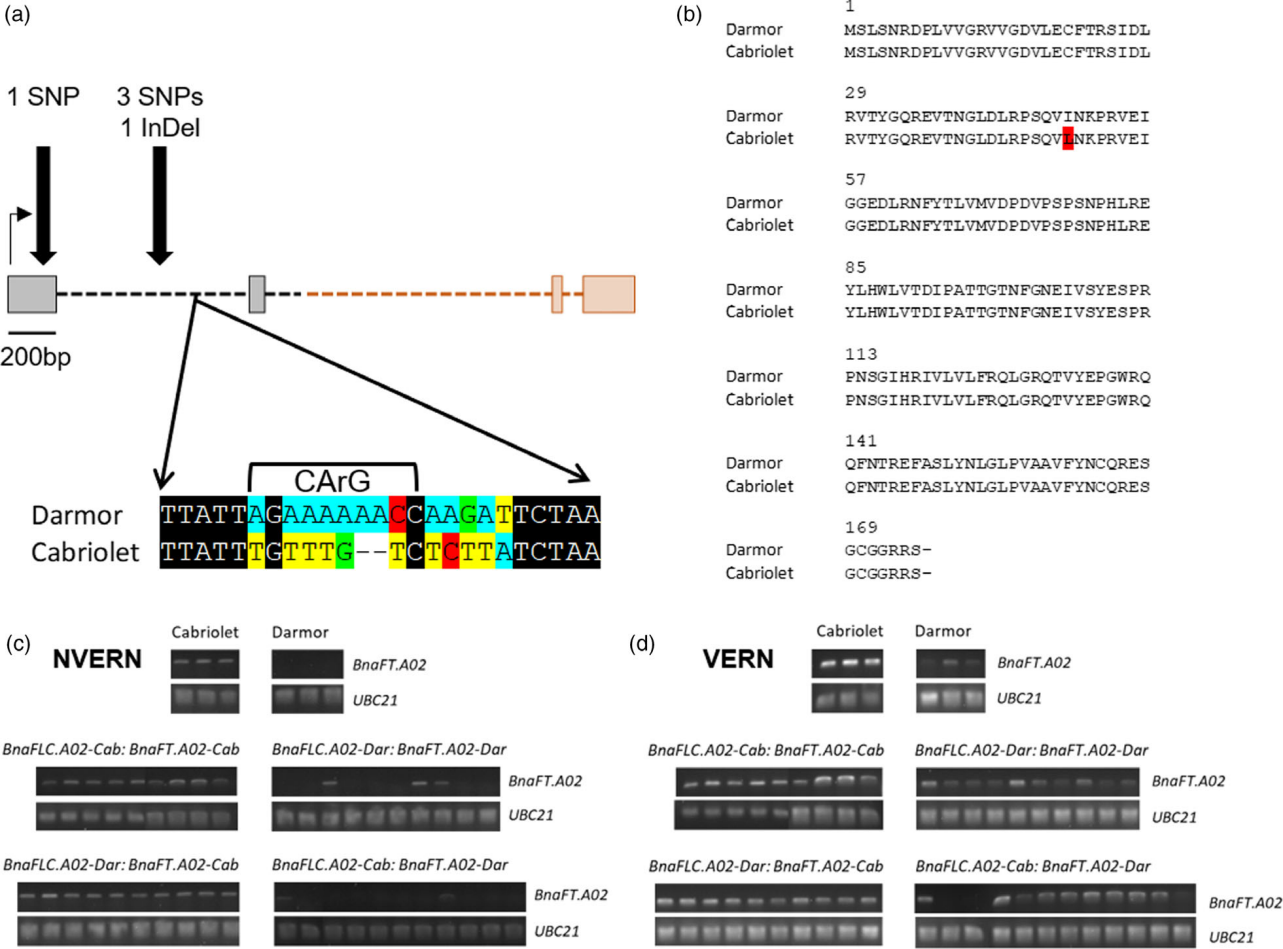
DNA sequence and gene expression variation of *BnaFT.A02* in Cabriolet and Darmor. (a) The polymorphisms identified at *BnaFT.A02* in Cabriolet compared with Darmor are highlighted with black arrows, grey boxes represent exons, black dashed lines represent introns, the black horizontal arrow represents the direction of transcription, and sequences not confirmed by capillary sequencing are highlighted in orange. Included is a zoom of the predicted CArG box motif sequence in Darmor and Cabriolet, conserved DNA sequences are coloured black, while polymorphisms are highlighted. (b) Comparison of the predicted amino acid sequence of BnaFT.A02 in Cabriolet and Darmor, amino acid substitution I48L is highlighted in red. (c) Qualitative expression analysis of *BnaFT.A02* in Cabriolet, Darmor and F_2_ lines genotyped for *BnaFLC.A02* and *BnaFT.A02* at 28 days after sowing and without vernalization treatment. Expression of *BnaFT.A02* is detectable when a band is present on the gel. (d) Qualitative expression analysis of *BnaFT.A02* in Cabriolet, Darmor and F_2_ lines genotyped for *BnaFLC.A02* and *BnaFT.A02* at 30 days under glasshouse conditions after a six‐week vernalization treatment. Expression of *BnaFT.A02* is detectable when a band is present on the gel.

*BnaFT.A02* was previously cloned by Wang *et al*. (2009) and, like *AtFT*, the gene is organized into four exons and three introns. Due to the presence of an AT rich insertion in intron 2 of *BnaFT.A02*, exons 1–2 of the gene were amplified and sequenced from the parental varieties. Alignment and analysis of this partial *BnaFT.A02* fragment from both varieties identified polymorphisms in both coding and non‐coding regions of the gene (Figure 6a). Compared with *BnaFT.A02* in Darmor (hereafter referred to as *BnaFT.A02‐Dar)*, 4 SNPs and 1 InDel were identified in the Cabriolet *BnaFT.A02* sequence (hereafter referred to as *BnaFT.A02‐Cab*; Figure 6a). One non‐synonymous SNP in exon 1 is predicted to cause an amino acid change from isoleucine to leucine (I48L; Figure 6b). The remaining SNPs and deletions were detected within intron 1, including sequence variation in Cabriolet that overlapped with the predicted CArG box motif sequence of *BnaFT.A02* where, in *A. thaliana*, the AtFLC protein binds and represses *AtFT* (Helliwell *et al*., 2006) (Figure 6a).

We assessed *BnaFT.A02* expression in Cabriolet, Darmor and in F_2_ lines (the same lines that had been genotyped for homozygosity at *BnaFLC.A02* and *BnaFT.A02* as described previously) with and without vernalization treatment using both qualitative and quantitative RT‐PCR (Figure 6c,d and Figure S7). Although lowly expressed, *BnaFT.A02‐Cab* expression was detectable in leaves at 28 days after sowing, while *BnaFT.A02‐Dar* expression was not detected (Figure 6c and Figure S7A). Under ambient temperature conditions, and without vernalization, *BnaFT.A02‐Cab* was consistently detectable up to 100 days after sowing, while *BnaFT.A02‐Dar* was not (Figure S7A). After a six‐week vernalization treatment and upon return to warm glasshouse conditions, the expression of *BnaFT.A02‐Cab* increased, while the expression of *BnaFT.A02‐Dar* remained low but detectable (Figures 6d and Figure S7B). To confirm that the absence of *BnaFT.A02* expression in Darmor was not due to a homeologous exchange, we quantified the expression of *BnaFT.C02* in both parent lines. Both *BnaFT.A02* and *BnaFT.C02* expression were activated after vernalization in Cabriolet, but the expression of both genes was not detectable in Darmor (Figure S7C). *BnaFT.A02* expression was assessed in a panel of F_2_ lines that could be grouped into four genotypes dependent on their homozygous alleles at *BnaFLC.A02* and *BnaFTA02* (Cab:Cab, Dar:Dar, Cab:Dar, Dar:Cab). *BnaFT.A02* expression was detectable at 28 days after sowing in F_2_ lines that were homozygous for *BnaFT.A02‐Cab* (Figure 6c). For most cases with lines carrying *BnaFT.A02‐Dar* (18 out of 21 lines), its expression was not detectable before vernalization (Figure 6c). In recombinant F_2_ lines, *BnaFT.A02‐Cab* expression was detectable regardless of the *BnaFLC.A02* allele present (Figure 6c). This suggests that, before vernalization, *BnaFT.A02‐Cab* expression is independent of the allele present at the *BnaFLC.A02* locus. After vernalization, expression of both alleles of *BnaFT.A02* was detectable in all F_2_ lines (Figure 6d). However, lines that carried the *BnaFLC.A02‐Cab* allele expressed *BnaFT.A02* at quantitatively higher levels compared with lines that carried the *BnaFLC.A02‐Dar* allele (Figure S7D,E).

## Discussion

Varieties of European winter oilseed rape are traditionally considered to have an obligate vernalization requirement, exhibiting an extended vegetative growth during the winter and flowering at the onset of spring. Here, we provide evidence that European winter oilseed rape varieties can exhibit an obligate or facultative vernalization requirement and we identify the same 10 Mbp genomic region on chromosome A02, which includes *BnaFT.A02* and *BnaFLC.A02*, as a candidate for controlling this requirement.

Previous reports (Chen *et al*., 2018; Nelson *et al*., 2014; Raman *et al*., 2016; Schiessl *et al*., 2015; Wu *et al*., 2019; Xu *et al*., 2016) have identified associations between regions on chromosome A02 and flowering time in *B. napus*. Variation at *BnaFLC.A02* and *BnaFT.A02* has been reported to contribute a significant proportion of variation in flowering time between crop types of oilseed rape (Chen *et al*., 2018; Wu *et al*., 2019), but here we report their role in flowering time within winter oilseed rape. Although our QTL encompassed a large genomic region and included several flowering time genes, genotypic data suggest that allelic variation at a region including *BnaFT.A02* contributed more strongly to flowering time without vernalization, while allelic variation at a region including *BnaFLC.A02* contributed to flowering time after vernalization.

We detected differences in the pre‐vernalization expression level of *BnaFLC.A02* between the early and late flowering varieties Cabriolet and Darmor, respectively. Striking differences in the expression profiles of both alleles of *BnaFLC.A02* were also detected in response to vernalization. The expression of the *BnaFLC.A02* in Cabriolet was stably silenced after six‐week vernalization, while the *BnaFLC.A02* allele in Darmor reactivated upon return to ambient temperatures. Variation in the epigenetic silencing of both *BnaFLC.A02* alleles highlight parallels with reports in *A. thaliana* and *Brassica oleracea* (Coustham *et al*., 2012; Irwin *et al*., 2016; Li *et al*., 2014). Variation in *FLC* silencing in both species (*AtFLC* and *BolFLC.C02*) is associated with sequence variation within non‐coding regions of the gene (Irwin *et al*., 2016; Li *et al*., 2014). Here, we report the presence of sequence polymorphisms at *BnaFLC.A02* within non‐coding intronic regions, while the BnaFLC.A02 protein of both alleles is predicted to be identical. The cis‐polymorphisms responsible for the variation in *BnaFLC.A02* expression are yet to be determined; however, based on reports in *A. thaliana* (Coustham *et al*., 2012; Li *et al*., 2014; Li *et al*., 2015; Questa *et al*., 2016) and due to conservation of *AtFLC* sequence between *A. thaliana* and *Brassica* sp. (Irwin *et al*., 2016; Schranz *et al*., 2002; Tadege *et al*., 2001; Wu *et al*., 2012; Xiao *et al*., 2013; Yuan *et al*., 2009; Zhao *et al*., 2010), it is reasonable to hypothesize that cis‐regulatory variation within intron 1 of *BnaFLC.A02* is likely to underpin the differential expression dynamics detected. As has been reported for winter accessions of *A. thaliana* (Coustham *et al*., 2012; Duncan *et al*., 2015; Li *et al*., 2014), variation in the length of cold required to induce epigenetically stable silencing of *BnaFLC.A02* is likely a major determinant of flowering time in European winter oilseed rape.

*BnaFT.A02* has previously been identified as a candidate gene for flowering time in *B. napus* (Long *et al*., 2007; Raman *et al*., 2016; Wang *et al*., 2009). Here, we have shown allelic variation at *BnaFT.A02* was associated with variation for flowering time in the absence of vernalization and is therefore similar to reports in Lupin (Nelson *et al*., 2017) and wheat (Yan *et al*., 2006). Although we were not able to amplify and sequence the whole *BnaFT.A02* gene from both varieties, we identified a non‐synonymous SNP in exon 1 of *BnaFT.A02* which distinguished Cabriolet from Darmor. This is predicted to encode an amino acid substitution (I48L) in the early flowering variety Cabriolet. The same amino acid substitution has been reported for a homologous gene *BraFT.A07* in *B. rapa* (Schiessl *et al*., 2017b; Zhang *et al*., 2015) but detected in a late flowering cultivar. As the I48L mutation involves substitution between two very similar amino acids and given it is associated with contrasting flowering time phenotypes in *B. rapa* and *B. napus*, we consider it unlikely that it confers the flowering time variation detected. However, this remains to be tested.

Vernalization length had a quantitative effect on *FT* expression; however, we detected differences between our early and late flowering varieties. *BnaFT.A02* was detectable in the early flowering variety Cabriolet before vernalization, and its expression increased after vernalization. In contrast, in the late flowering variety Darmor, *BnaFT.A02* expression was not detectable by RT‐PCR before vernalization, and although detectable after vernalization, the gene was expressed at low levels as determined by qRT‐PCR. A previous report in *B. rapa* identified an insertion within intron 2 of *BraFT.A07* which was associated with late flowering due to failed transcription of *BraFT.A07* (Zhang *et al*., 2015). We detected the presence of polymorphisms at the CArG box motif sequence within intron 1 of *BnaFT.A02* in Cabriolet compared with Darmor. In *A. thaliana*, the AtFLC protein binds directly with the CArG box motif sequence within intron 1 of *AtFT* to inhibit its expression (Helliwell et al., 2006). It is therefore plausible that variation at cis‐regulatory regions of *BnaFT.A02*, such as the polymorphisms detected at the CArG box motif, has resulted in a lack of FLC protein binding in intron 1 of *BnaFT.A02* leading to low but detectable expression of *BnaFT.A02* in Cabriolet prior to vernalization.

Genotypic data confirmed that the allelic combination *BnaFLC.A02‐Cab* and *BnaFT.A02‐Cab* conferred the earliest flowering, while *BnaFLC.A02‐Dar* and *BnaFT.A02‐Dar* conferred the latest flowering, with and without vernalization. Analysis of recombinant F_2_ lines segregating for *BnaFLC.A02* and *BnaFT.A02* indicated the phenotypic effect of both genes was dependent on genotype and vernalization treatment. *BnaFLC.A02‐Cab* when in combination with *BnaFT.A02‐Dar* conferred late flowering in the absence of vernalization, but early flowering after vernalization. In contrast, *BnaFLC.A02‐Dar* when in combination with *BnaFT.A02‐Cab* conferred early flowering in the absence of vernalization, but late flowering after vernalization. This suggests that *BnaFT.A02* was more strongly associated with earliness of flowering in the absence of vernalization and that *BnaFLC.A02* played a more dominant role in flowering after vernalization. Analysis of expression of both genes in recombinant F_2_ lines demonstrated that *BnaFT.A02* and *BnaFLC.A02* acted independently from one another before vernalization. Expression of the Cabriolet allele of *BnaFT.A02* was detectable before vernalization treatment, regardless of *BnaFLC.A02* allele, and the Darmor allele of *BnaFLC.A02* exhibited reactivation of expression after vernalization independent of *BnaFT.A02* allele. We hypothesize polymorphisms that affect the expression dynamics of both genes underpin the association with flowering time variation under vernalized and non‐vernalized growth conditions.

Cis‐variation at orthologues of *AtFLC* and *AtFT* in *B. napus* is likely to have a major influence on gene expression and ultimately the flowering time phenotype of the crop. Like in *A. thaliana*, cis‐variation resulting in changes to transcription factor binding sites may have played an important role in flowering time evolution in *B. napus*. We hypothesize plant breeding for varieties adapted to varying winter climates has selected for variation at *BnaFLC.A02* and *BnaFT.A02* in European winter oilseed rape adapted to different environmental conditions. This knowledge will allow for the selection of alleles of flowering time regulators that alter the vernalization requirement of oilseed rape, informing the generation of new varieties with adapted flowering times and improved yields.

## Methods

### Plant materials and growth conditions

An F_2_ population generated from a reciprocal cross between two inbred lines derived from the winter oilseed rape varieties Cabriolet and Darmor (both sourced from the oilseed rape genetic improvement network (OREGIN) *B. napus* diversity fixed foundation set population; http://www.herts.ac.uk/oregin) were used in the present study. Five heterozygous F_1_ siblings from each reciprocal cross were selected and carried forward to the F_2_ generation by self‐fertilization.

Seeds were sown directly onto soil (Levington F2 compost, 600 L peat, 100 L 4 mm grit, 196 g ‘Exemptor’ chloronicotinyl insecticide) and grown under glasshouse conditions (16‐h light/8‐h dark, 600 W HPS lamps provided supplementary lighting when required, 18 °C day temperature, 15 °C night temperature and 70% humidity) for 28 days before receiving one of two vernalization treatments; six‐week vernalization (VERN) or no vernalization (NVERN). For the NVERN treatment, plants were grown for 28 days under glasshouse conditions. For the VERN treatment, after 28 days’ growth under glasshouse conditions, plants were transferred to a vernalization chamber (5 °C, 8‐h light/16‐h dark, 70% humidity) for six weeks. For both vernalization treatments, a total F_2_ population of 720 lines (72 randomly selected seed from each of the ten F_1_ plants) in addition to 12 plants each of Cabriolet and Darmor were grown and assessed for flowering time variation. Sowing was staggered so that all plants from the NVERN and VERN treatments were transferred to a concrete floored poly‐tunnel (Keder house; https://www.kedergreenhouse.co.uk/) at the John Innes Centre, Norwich on the 5^th^ and 6^th^ April 2017, respectively. Plants received no supplementary heating or lighting, were transplanted to 1 L pots and arranged in a randomized complete block design containing four blocks at a density of 36 plants/m^2^. Plants were watered twice daily by automatic irrigation and chemically sprayed when required. Temperature and humidity within the poly‐tunnel were recorded by Tinytag (Gemini Data Loggers, Chichester, UK) at 30‐min intervals for the duration of the experiment.

Flowering time was measured as the number of days to opening of the first flower (BBCH60 according to Meier *et al*. (2009)). Flowering time measurements commenced on the date of transfer to the poly‐tunnel (28 days after sowing for NVERN and 70 days after sowing for VERN) and continued for 170 days. All plants that had no open flowers at the end of the experiment were given a did not flower (DNF) score of 170 days. Statistical analysis was performed using GenStat 18^th^ Edition (VSN International, Hemel Hempstead, UK).

### Construction of bulks and Illumina sequencing

To identify sequence variants between the parents of the F_2_ population, DNA was extracted from leaf tissue from both parent plants of the reciprocal cross, Cabriolet and Darmor, and sequenced by whole‐genome sequencing. The DNA bulks were generated based on the flowering time measurements obtained for the F_2_ population. Leaf material was pooled prior to DNA extraction, and each pool contained a 1cm leaf disc taken from lines that represented the phenotypic extremes of the population (approximately 5% of lines from both tail ends of the flowering time distribution). The number of F_2_ lines in each bulk is listed in Table S1. A total of four DNA bulks; early flowering bulk for VERN treatment (VERN_EARLY), late flowering bulk for VERN treatment (VERN_LATE), early flowering bulk for the NVERN treatment (NVERN_EARLY) and late flowering bulk for the NVERN treatment (NVERN_EARLY), were sequenced by whole‐genome sequencing.

DNA was extracted using a CTAB‐based method and samples prepared for Illumina sequencing by Novogene Co., Ltd., Hong Kong (https://www.novogene.com). DNA libraries were prepared using TruSeq DNA Sample HT Sample Preparation Kit (Illumina, San Diego, CA) following the manufacturer's recommendations. 1µg of DNA was fragmented using Covaris cracker, end‐repaired and adapter‐ligated. After PCR enrichment, DNA libraries were purified (AMPure XP system) and analysed for size distribution by Agilent2100 Bioanalyzer. The DNA libraries were sequenced on Illumina HiSeq X platform (Illumina Inc.) to generate 300‐base paired‐end reads at an average of 30x coverage. Sample quality control, library construction and sequencing were performed by Novogene Co., Ltd., HK.

### Construction of sequence assemblies

The Darmor‐*bzh B. napus* genome sequence (Chalhoub *et al*., 2014, Genome Assembly: AST_PRJEB5043_v1, plants.ensembl.org) was used as a reference. The sequencing reads from the six samples (Cabriolet, Darmor, VERN_EARLY, VERN_LATE, NVERN_EARLY, NVERN_LATE) were aligned to the Darmor‐*bzh* genome reference sequence using Bowtie‐2 v2.2.3 (Langmead *et al*., 2009) to create six separate alignment files in Sequence Alignment/Map (SAM) format which were converted to BAM (.bam) file format using SAMtools v1.5 (Li *et al*., 2009). For stringency, sequence data from non‐uniquely mapped reads were excluded from the alignment by filtering for the ‘‐q 42’ parameter in SAMtools. A sequence PileUp was then generated using the ‘mpileup’ command in SAMtools (Li *et al*., 2009).

Variants (SNPs and small Insertion/Deletions or InDels (<9bp)) were called in Cabriolet compared with Darmor, and their genomic position was assigned according to the Darmor‐*bzh* reference genome (Chalhoub *et al*., 2014). High confidence variants were called when the read depth was greater than, or equal to, 20 and the alternative sequence was found in ≥ 95% of Cabriolet sequence reads. All other genomic positions were excluded from further analysis. Genomic positions that were genetically identical, that is no alternative sequence was detected in Cabriolet compared with Darmor, were also excluded.

### Calculation of SNP index

SNP index values were calculated for the bulks according to Abe *et al*. (2012) and Takagi *et al*. (2013). In this study, the SNP index was a measure of the proportion of sequencing reads at a given variant position that differed from our chosen reference variety, Darmor. At each variant position where the read depth was ≥20 in both bulks, the proportion of total reads matching the alternative variant found in Cabriolet was calculated to give a SNP index value. Although the sequence read depth filtering parameter used in this study was stringent, a relatively even distribution of genomic positions was included in the analysis (Figure S8). A SNP index value of 1 indicated all sequencing reads at that position were derived from Cabriolet, while a SNP index value of 0 indicated all sequencing reads were derived from Darmor. A SNP index value equal to 0.5, however, indicated equal contribution of alleles from both parents, Cabriolet and Darmor, in the DNA bulk. A deviation in SNP index value away from 0.5 indicated a bias in the genetic contribution of both parents between the DNA bulks. Large deviations in SNP index values from 0.5 would identify associations between a genomic region and the phenotypic differences between the DNA bulks. A delta (Δ) SNP index value was then calculated by subtracting the SNP index of the early bulk from the SNP index of the late bulk at each variant position. To reduce ambiguity introduced by sequencing error, SNP index values that were <0.3 in both bulks were excluded (as per Takagi *et al*., 2013). Regions of the genome representing the top 1% of absolute ΔSNP index values were considered to be strongly associated with flowering time. The code used for this analysis is available open source (https://github.com/marc-jones/brassica-napus-bulk-segregant).

### Validation of alleles within the QTL region

To validate the QTL region identified on chromosome A02, allele‐specific KASP (Kompetitive allele‐specific PCR) primers (LGC Genomics, https://www.biosearchtech.com/services/genotyping-services) were designed to target five SNPs within a 6Mbp region located between base pair positions 136 553 and 6 375 504 on chromosome A02 (Table S2). The genomic positions for each SNP were according to the Darmor‐*bzh* reference genome and included SNPs within orthologues of the flowering time genes *AtFLC* (SNP FLC‐136553 within *BnaFLC.A02*) and *AtFT* (SNP FT‐6375504 within *BnaFT.A02*). DNA was extracted from the 94 earliest and 94 latest F_2_ lines under both NVERN and VERN treatments, in addition to the parental lines Cabriolet and Darmor, and assayed for SNP genotype using the KASP genotyping chemistry according to the manufacturer's instructions (LGC Genomics, Hoddesdon, UK).

### Sequence analysis of *BnaFLC.A02* and *BnaFT.A02*

To assess for DNA polymorphisms, sequences for *BnaFLC.A02* and *BnaFT.A02* were extracted from the Cabriolet and Darmor sequence PileUp and aligned against the Darmor‐*bzh BnaFLC.A02* and *BnaFT.A02* sequences (Chalhoub *et al*., 2014), downloaded from plants.ensembl.org. Sequence polymorphisms were identified, and amino acid sequence changes were predicted using AlignX (Vector NTI Advance®, Invitrogen^TM^, now Thermo Fisher Scientific, https://www.thermofisher.com). To confirm the presence of the polymorphisms in Cabriolet and Darmor, primers were designed to amplify and sequence regions of the *BnaFLC.A02* and *BnaFT.A02* genes from both varieties (Table S3). DNA was isolated from both varieties using the Edwards DNA extraction method (Edwards *et al*., 1991), and PCR was performed using AmpliTaq Gold^TM^ DNA polymerase (Applied Biosystems^TM^, now Thermo Fisher Scientific) according to the manufacturer's instructions with an annealing temperature of 58 °C. PCR products were prepared for sequencing using the Big Dye V3.1^TM^ terminator protocol (Applied Biosystems^TM^, now Thermo Fisher Scientific), and capillary sequencing was performed by Eurofins Genomics, EU. Sequences were aligned and analysed using AlignX.

### Quantitative expression analysis of *BnaFLC.A02* and *BnaFT.A02*

For quantitative expression analysis of *BnaFLC.A02* and *BnaFT.A02*, leaf material was taken from the newest expanded leaf of Cabriolet, Darmor and F_2_ plants genotyped and determined homozygous by KASP assay for *BnaFLC.A02* and *BnaFT.A02* before (NV), at the end (T0) and after (T16, T30) a six‐week vernalization treatment. Leaf material was sampled at the same time of day at the ¾ point of the photoperiod regime to account for photoperiodic effects on gene expression. Total RNA was extracted from individual leaf samples using the E.Z.N.A.® Plant RNA Kit (Omega Bio‐tek, Georgia, USA) and contaminating DNA were removed using the on‐column RNase‐free DNase Set I (Omega Bio‐tek) according to the manufacturer's instructions. Two micrograms RNA was converted to cDNA using Superscript^TM^ III Reverse Transcriptase (Invitrogen^TM^, now Thermo Fisher Scientific) and gene‐specific reverse primers (Table S4) according to the manufacturer's instructions. qRT‐PCR was performed using LightCycler® 480 SYBR Green I Master Mix on the LightCycler® 480 II instrument (both Roche, www.roche.com). Second‐derivative maximum values were calculated using the LightCycler® Software to give absolute expression values. Expression values of *BnaFLC.A02* and *BnaFT.A02* were normalized to the internal reference gene *UBC21* (Orsel *et al*., 2014) using the ΔΔCT method (Livak and Schmittgen, 2001). For qualitative expression analysis of *BnaFT.A02*, the same cDNA was amplified by PCR using AmpliTaq Gold^TM^ DNA polymerase (Thermo Fisher Scientific) and PCR products were visualized by agarose gel electrophoresis.

## Accession numbers

All raw sequence reads for Cabriolet, Darmor and the four DNA bulks have been deposited in the European Nucleotide Archive under PRJEB33550. *BnaFLC.A02* and *BnaFT.A02* sequence data for varieties Cabriolet and Darmor can be found under GenBank accession numbers MN218571, MN218572, MN218573 and MN218574.

## Conflict of interest

The authors declare no conflict of interest.

## Author contributions

E.H.T., J.A.I. and C.D. conceived the project. All *in vivo* experiments were conducted by E.H.T. D.M.J. performed all bioinformatics associated with the QTL‐seq analysis. Z. H. and I.B. performed the HE analysis. M.T. contributed to bulk sampling design and conducted initial bioinformatics analysis. R.W. assisted with population development and experimental design. E.H.T., J.A.I. and C.D. wrote the manuscript. All authors read and approved the final manuscript.

## Supporting information


**Figure S1** Temperature and humidity recorded within the Keder plastic poly‐tunnel during the 2017 flowering time phenotyping analysis of F_2_ lines at JIC.
**Figure S2** Frequency distributions of sequence variants (SNPs and small InDels) detected in Darmor compared with Darmor‐*bzh*. Each chromosome is plotted separately, and the frequency of variants detected are plotted by genome order.
**Figure S3** Frequency distributions of sequence variants (SNPs and small InDels) detected in Cabriolet compared with Darmor. Each chromosome is plotted separately, and the frequency of variants detected are plotted by genome order.
**Figure S4** No sequence variants (SNPs and small InDels) are detected in Cabriolet compared with Darmor at the major flowering time genes *BnaFRI.A03* and *BnaFLC.A10*. (A) The relative position of *BnaFRI.A03* on chromosome A03 is plotted with the closest sequence variant found up‐ and down‐stream highlighted with a red arrow. (B) The relative position of *BnaFLC.A10* on chromosome A10 is plotted with the closest sequence variant found up‐ and down‐stream highlighted with a red arrow.
**Figure S5** Visualization of homeologous genome exchanges in Cabriolet and Darmor based on DNA resequencing.
(A & B): The relative redundancy of coverage of A and C genome homeologous gene pairs is represented in CMYK colour space, with cyan component representing coverage of the *Brassica* A genome copy and magenta component representing coverage of the *Brassica* C genome copy.
(A) Genome‐wide homeologous genome exchanges in Cabriolet and Darmor. The gene pairs are plotted in Brassica C genome order (chromosomes denoted C1 to C9).
(B) Homeologous exchanges present on chromosome A02/C02. The gene pairs are plotted in Brassica chromosome C02 gene order, the relative position of *BnaFLC.A02/C02* and *BnaFT.A02/C02* gene pairs are highlighted.
**Figure S6** Expression of *BnaFLC.A02* varies between Cabriolet and Darmor before and after vernalisation.
(A‐D) Normalised expression of *BnaFLC.A02* in Cabriolet, Darmor, and genotyped F_2_ individuals as measured by quantitative RT‐PCR before (NV) and after vernalisation (6WT0, 6WT16, 6WT30). The expression levels, normalised to *UBC21*, detected in each line plant are plotted here.
(A) Normalised expression of *BnaFLC.A02* detected in 3 Cabriolet, 3 Darmor and 10 F_2_ individuals with genotypic combination BnaFLC.A02‐Dar/BnaFT.A02‐Dar.
(B) Normalised expression of *BnaFLC.A02* detected in 3 Cabriolet, 3 Darmor and 10 F_2_ individuals with genotypic combination BnaFLC.A02‐Dar/BnaFT.A02‐Cab.
(C) Normalised expression of *BnaFLC.A02* detected in 3 Cabriolet, 3 Darmor and 10 F_2_ individuals with genotypic combination BnaFLC.A02‐Cab/BnaFT.A02‐Dar.
(D) Normalised expression of *BnaFLC.A02* detected in 3 Cabriolet, 3 Darmor and 10 F_2_ individuals with genotypic combination BnaFLC.A02‐Cab/BnaFT.A02‐Cab.
**Figure S7** Expression of *BnaFT* is detected in Cabriolet, but not detectable in Darmor.
(A) Normalised expression of *BnaFT.A02* in Cabriolet and Darmor over time and under ambient temperature conditions as measured by quantitative RT‐PCR error bars denote one standard error around the mean calculated from at least three biological replicates, T= days from sowing.
(B) Normalised expression of *BnaFT.A02* in Cabriolet and Darmor as measured by quantitative RT‐PCR before (NV) and after vernalisation (6WT0, 6WT16, 6WT30), error bars denote one standard error around the mean calculated from three biological replicates.
(C) Normalised expression of *BnaFT.C02* in Cabriolet and Darmor as measured by quantitative RT‐PCR before (NV) and after vernalisation (6WT0, 6WT16, 6WT30), error bars denote one standard error around the mean calculated from three biological replicates.
(D) Normalised expression of *BnaFT.A02* in F_2_ lines genotyped for *BnaFLC.A02* and *BnaFT.A02* as measured by quantitative RT‐PCR before (NV) and after vernalisation (6WT0, 6WT16, 6WT30), error bars denote one standard error around the mean calculated from at least three biological replicates.
(E) Normalised expression of *BnaFT.A02* in F_2_ lines genotyped for *BnaFLC.A02* and *BnaFT.A02* as measured by quantitative RT‐PCR before (NV) and after vernalisation (6WT0, 6WT16, 6WT30), error bars denote one standard error around the mean calculated from at least three biological replicates.
**Figure S8** The distribution of genomic positions included in the QTL‐seq analysis. Each chromosome is plotted as separate histograms of the frequency of genomic positions with read depth coverage of more than 20 reads and included in SNP and ΔSNP indices calculation.

**Table S1** Summary of flowering time and Illumina sequencing data of parent lines and bulks for treatments VERN and NVERN.
**Table S2** KASP primers used for validation of QTL region.
**Table S3** Primers used to amplify and sequence *BnaFLC.A02* and *BnaFT.A02*.
**Table S4** Primers used for quantitative RT‐PCR analysis of *BnaFLC.A02* and *BnaFT.A02*.

Supplementary Material
