# Supplementary material for: QTL‐seq identifies BnaFT.A02 and BnaFLC.A02 as candidates for variation in vernalization requirement and response in winter oilseed rape (Brassica napus)
Source: Plant Biotechnol J. 2020 Jun 23;18(12):2466–81. doi: 10.1111/pbi.13421 (PMC7680531; doi:10.1111/pbi.13421)
Supplement: Supplementary file 1 — Figure S1 Temperature and humidity recorded within the Keder plastic poly‐tunnel during the 2017 flowering time phenotyping analysis of F2 lines at JIC. Figure S2 Frequency distributions of sequence variants (SNPs and small InDels) detected in Darmor compared with Darmor‐bzh. Each chromosome is plotted separately, and the frequency of variants detected are plotted by genome order. Figure S3 Frequency distributions of sequence variants (SNPs and small InDels) detected in Cabriolet compared with Darmor. Each chromosome is plotted separately, and the frequency of variants detected are plotted by genome order. Figure S4 No sequence variants (SNPs and small InDels) are detected in Cabriolet compared with Darmor at the major flowering time genes BnaFRI.A03 and BnaFLC.A10. (A) The relative position of BnaFRI.A03 on chromosome A03 is plotted with the closest sequence variant found up‐ and down‐stream highlighted with a red arrow. (B) The relative position of BnaFLC.A10 on chromosome A10 is plotted with the closest sequence variant found up‐ and down‐stream highlighted with a red arrow. Figure S5 Visualization of homeologous genome exchanges in Cabriolet and Darmor based on DNA resequencing. (A & B): The relative redundancy of coverage of A and C genome homeologous gene pairs is represented in CMYK colour space, with cyan component representing coverage of the Brassica A genome copy and magenta component representing coverage of the Brassica C genome copy. (A) Genome‐wide homeologous genome exchanges in Cabriolet and Darmor. The gene pairs are plotted in Brassica C genome order (chromosomes denoted C1 to C9). (B) Homeologous exchanges present on chromosome A02/C02. The gene pairs are plotted in Brassica chromosome C02 gene order, the relative position of BnaFLC.A02/C02 and BnaFT.A02/C02 gene pairs are highlighted. Figure S6 Expression of BnaFLC.A02 varies between Cabriolet and Darmor before and after vernalisation. (A‐D) Normalised expression of BnaFLC.A02 in Cabriolet, Darm [file PBI-18-2466-s002.pdf]

Figure S1

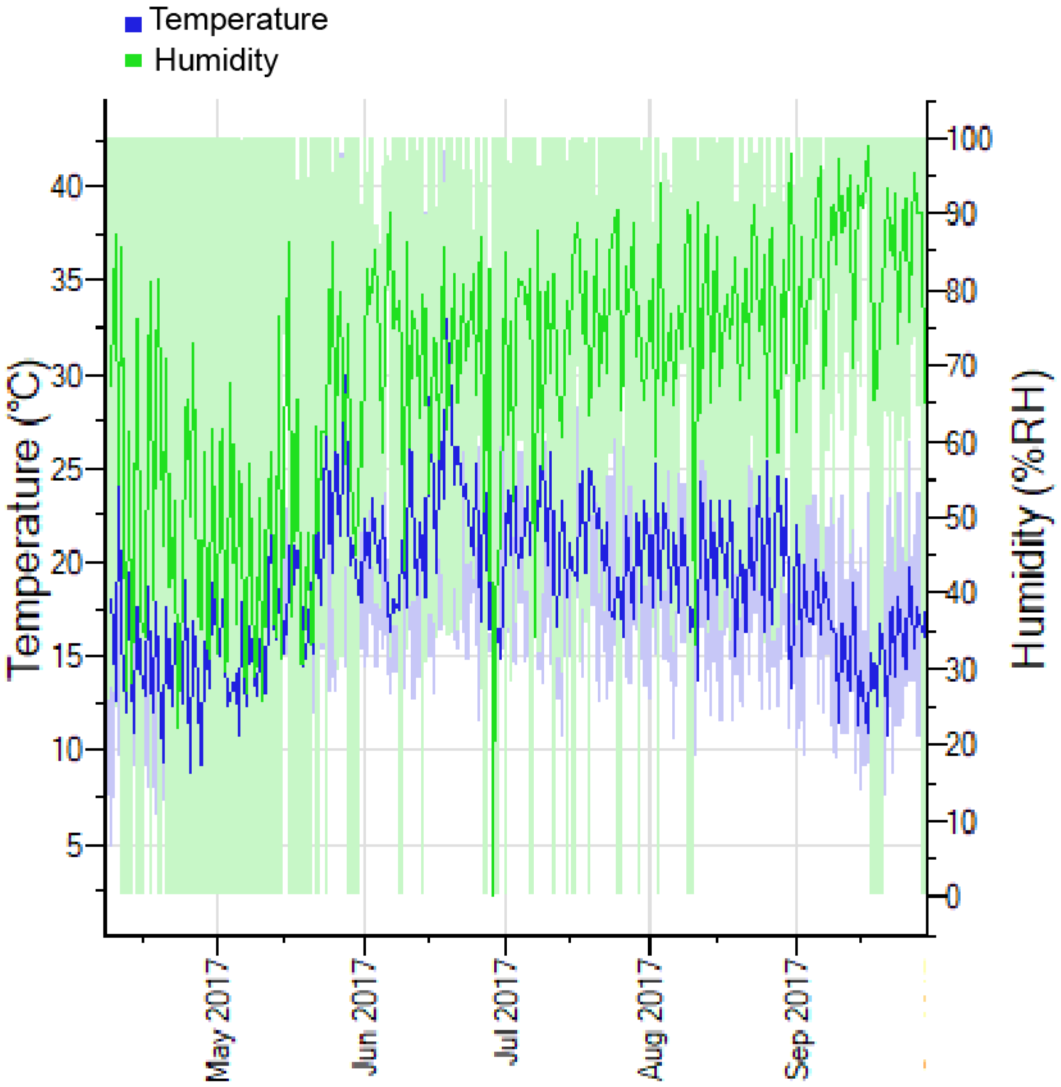
Temperature
Humidity
Temperature (°C)
40
35
30
25
20
15
10
5
Humidity (%RH)
100
90
80
70
60
50
40
30
20
10
0
May 2017
Jun 2017
Jul 2017
Aug 2017
Sep 2017

Figure S2

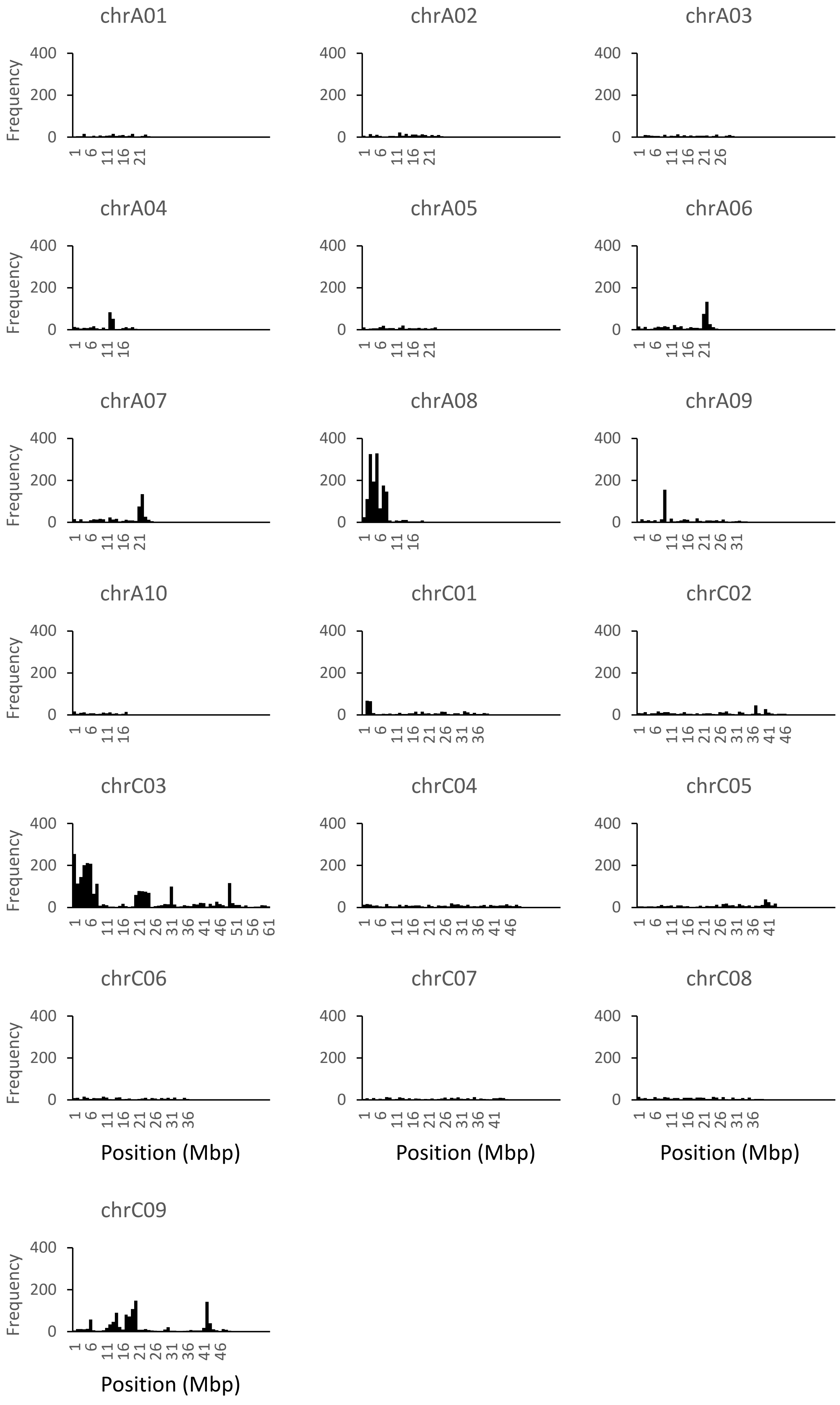
chrA01
chrA02
chrA03
chrA04
chrA05
chrA06
chrA07
chrA08
chrA09
chrA10
chrC01
chrC02
chrC03
chrC04
chrC05
chrC06
chrC07
chrC08
chrC09
Frequency
0
200
400
Position (Mbp)

Figure S3

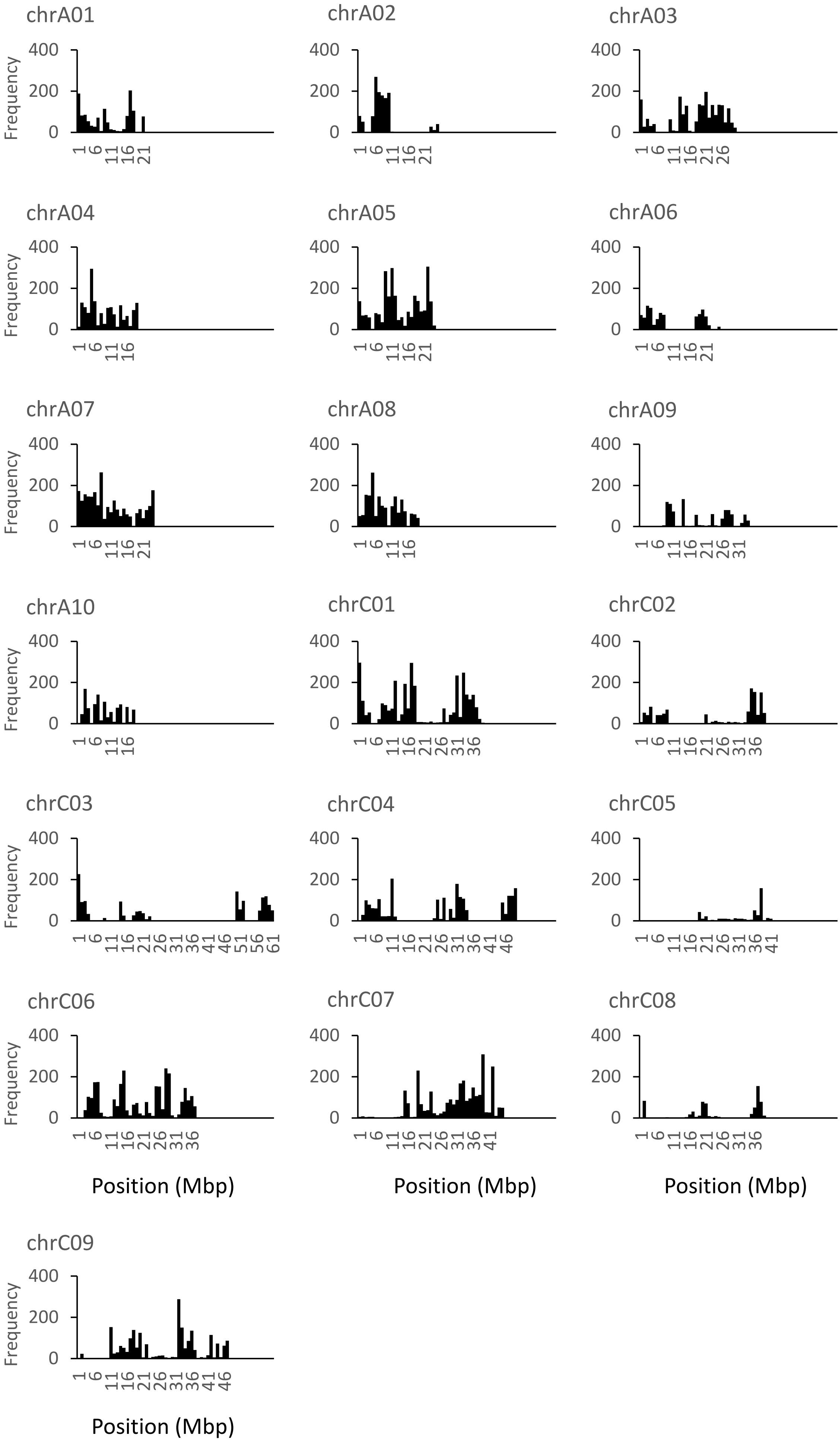

chrA01
chrA02
chrA03
chrA04
chrA05
chrA06
chrA07
chrA08
chrA09
chrA10
chrC01
chrC02
chrC03
chrC04
chrC05
chrC06
chrC07
chrC08
chrC09
Frequency
400
200
0
Position (Mbp)

Figure S4

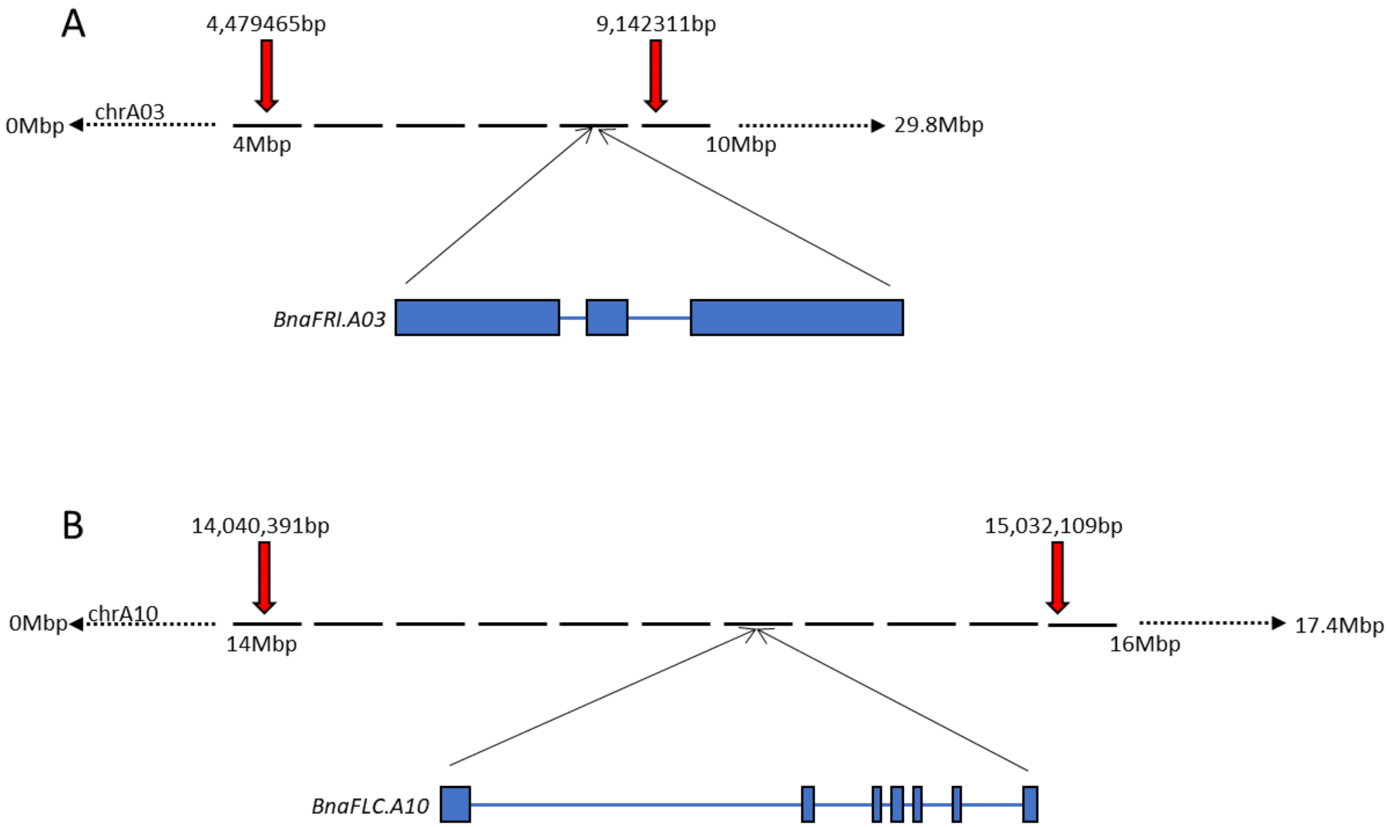
A
4,479465bp
9,142311bp
0Mbp
chrA03
29.8Mbp
4Mbp
10Mbp
BnaFRI.A03
B
14,040,391bp
15,032,109bp
0Mbp
chrA10
17.4Mbp
14Mbp
16Mbp
BnaFLC.A10

Figure S5

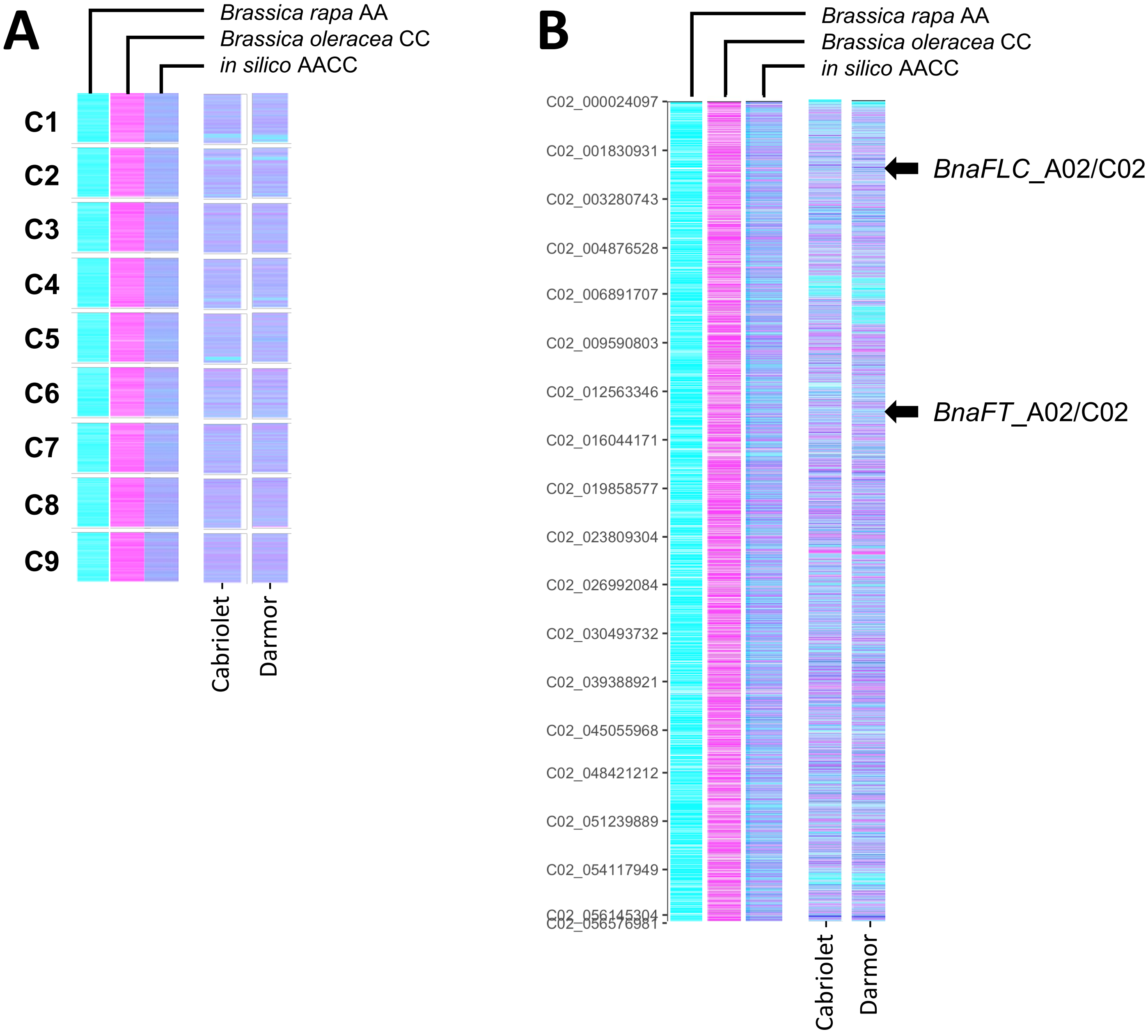

A
Brassica rapa AA
Brassica oleracea CC
in silico AACC
C1
C2
C3
C4
C5
C6
C7
C8
C9
Cabriolet
Darmor
B
Brassica rapa AA
Brassica oleracea CC
in silico AACC
C02_000024097
C02_001830931
C02_003280743
C02_004876528
C02_006891707
C02_009590803
C02_012563346
C02_016044171
C02_019858577
C02_023809304
C02_026992084
C02_030493732
C02_039388921
C02_045055968
C02_048421212
C02_051239889
C02_054117949
C02_056145304
C02_056576981
BnaFLC_A02/C02
BnaFT_A02/C02
Cabriolet
Darmor

Figure S6

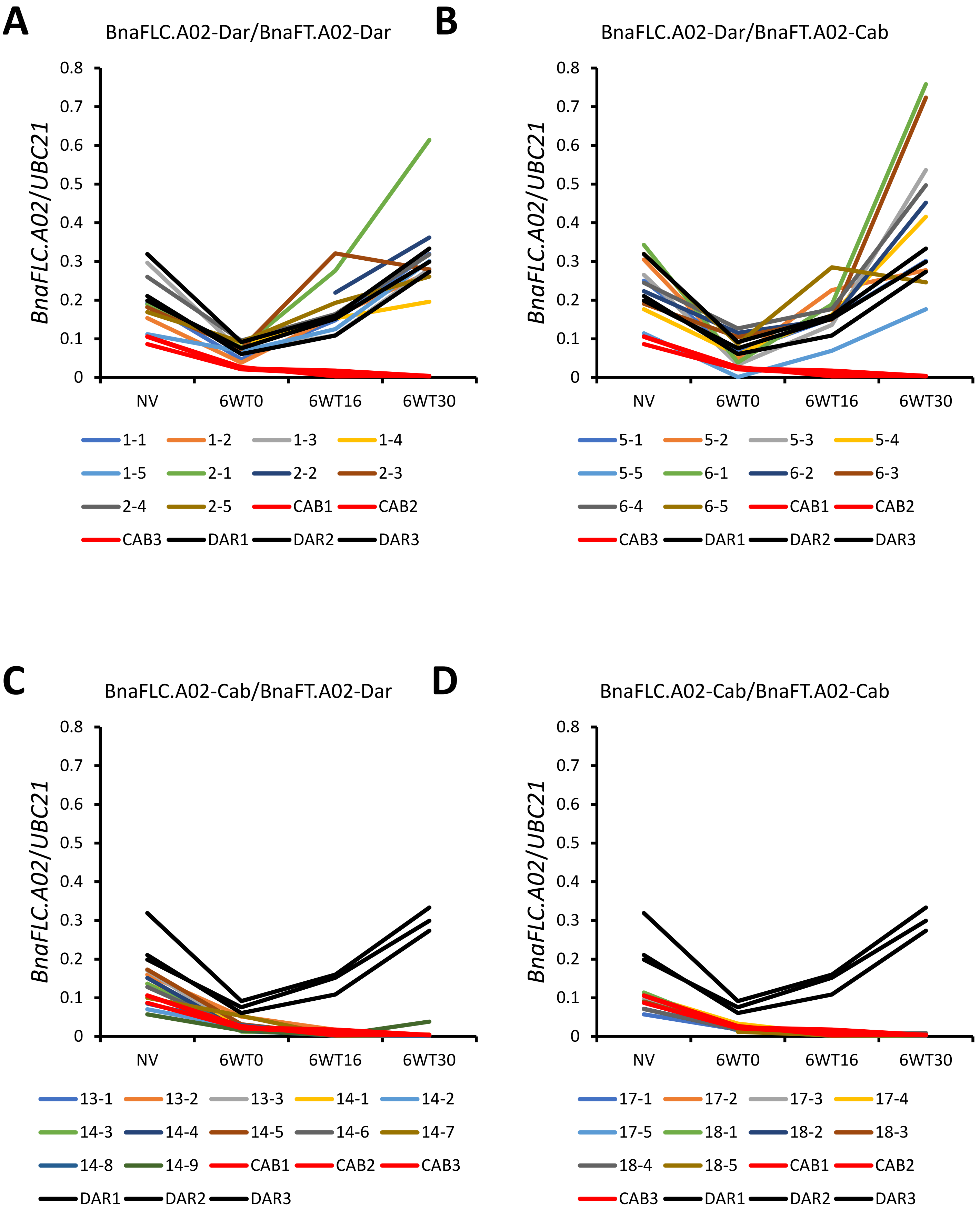
A
BnaFLC.A02-Dar/BnaFT.A02-Dar
B
BnaFLC.A02-Dar/BnaFT.A02-Cab
C
BnaFLC.A02-Cab/BnaFT.A02-Dar
D
BnaFLC.A02-Cab/BnaFT.A02-Cab
BnaFLC.A02/UBC21
0.8
0.7
0.6
0.5
0.4
0.3
0.2
0.1
0
NV
6WT0
6WT16
6WT30
1-1 1-2 1-3 1-4 1-5 2-1 2-2 2-3 2-4 2-5 CAB1 CAB2 CAB3 DAR1 DAR2 DAR3
5-1 5-2 5-3 5-4 5-5 6-1 6-2 6-3 6-4 6-5 CAB1 CAB2 CAB3 DAR1 DAR2 DAR3
13-1 13-2 13-3 14-1 14-2 14-3 14-4 14-5 14-6 14-7 14-8 14-9 CAB1 CAB2 CAB3 DAR1 DAR2 DAR3
17-1 17-2 17-3 17-4 17-5 18-1 18-2 18-3 18-4 18-5 CAB1 CAB2 CAB3 DAR1 DAR2 DAR3

Figure S7

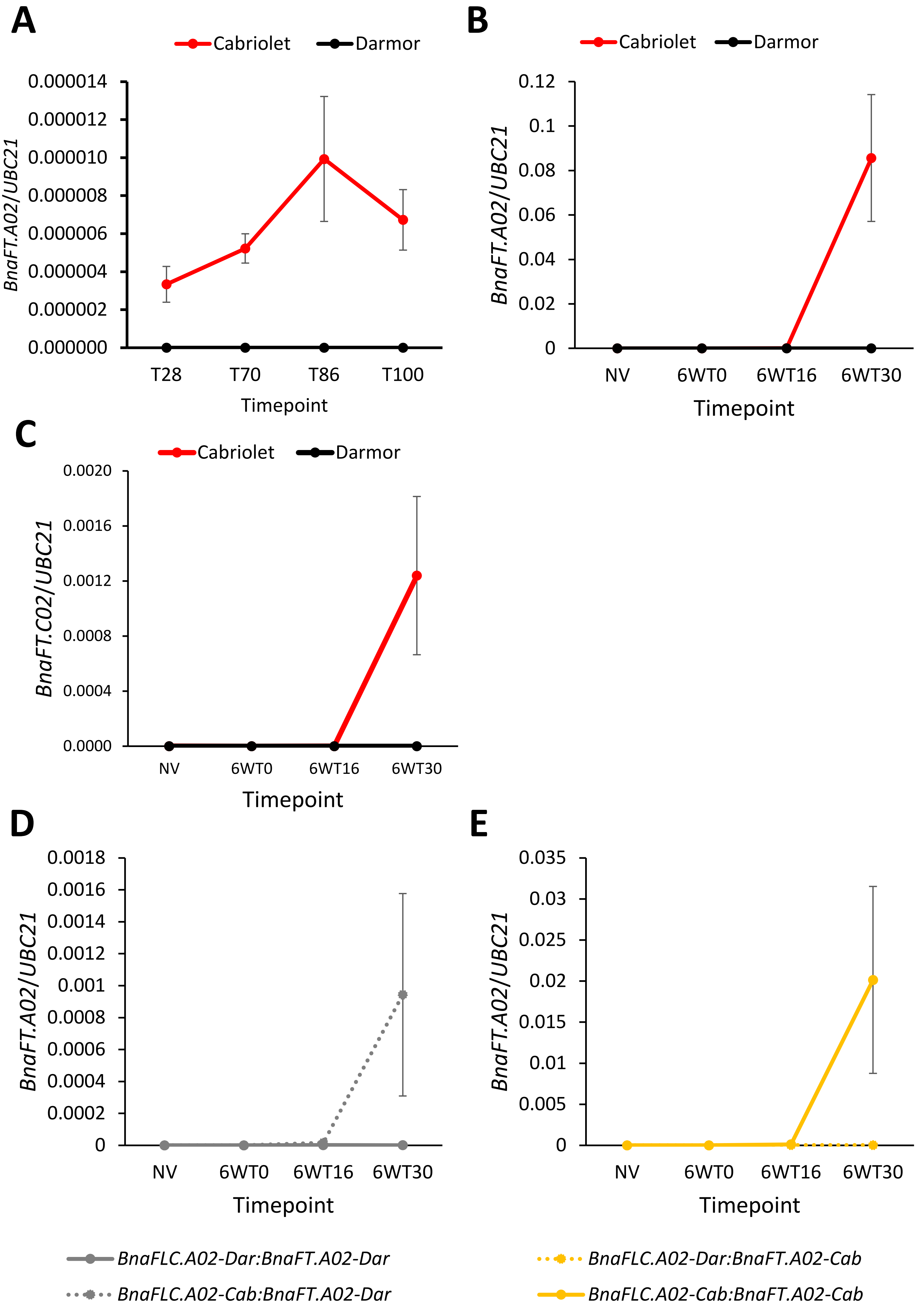
A
Cabriolet
Darmor
BnaFT.A02/UBC21
0.000014
0.000012
0.000010
0.000008
0.000006
0.000004
0.000002
0.000000
T28
T70
T86
T100
Timepoint
B
Cabriolet
Darmor
BnaFT.A02/UBC21
0.12
0.1
0.08
0.06
0.04
0.02
0
NV
6WT0
6WT16
6WT30
Timepoint
C
Cabriolet
Darmor
BnaFT.C02/UBC21
0.0020
0.0016
0.0012
0.0008
0.0004
0.0000
NV
6WT0
6WT16
6WT30
Timepoint
D
BnaFT.A02/UBC21
0.0018
0.0016
0.0014
0.0012
0.001
0.0008
0.0006
0.0004
0.0002
0
NV
6WT0
6WT16
6WT30
Timepoint
E
BnaFT.A02/UBC21
0.035
0.03
0.025
0.02
0.015
0.01
0.005
0
NV
6WT0
6WT16
6WT30
Timepoint
BnaFLC.A02-Dar:BnaFT.A02-Dar
BnaFLC.A02-Cab:BnaFT.A02-Dar
BnaFLC.A02-Dar:BnaFT.A02-Cab
BnaFLC.A02-Cab:BnaFT.A02-Cab

Figure S8

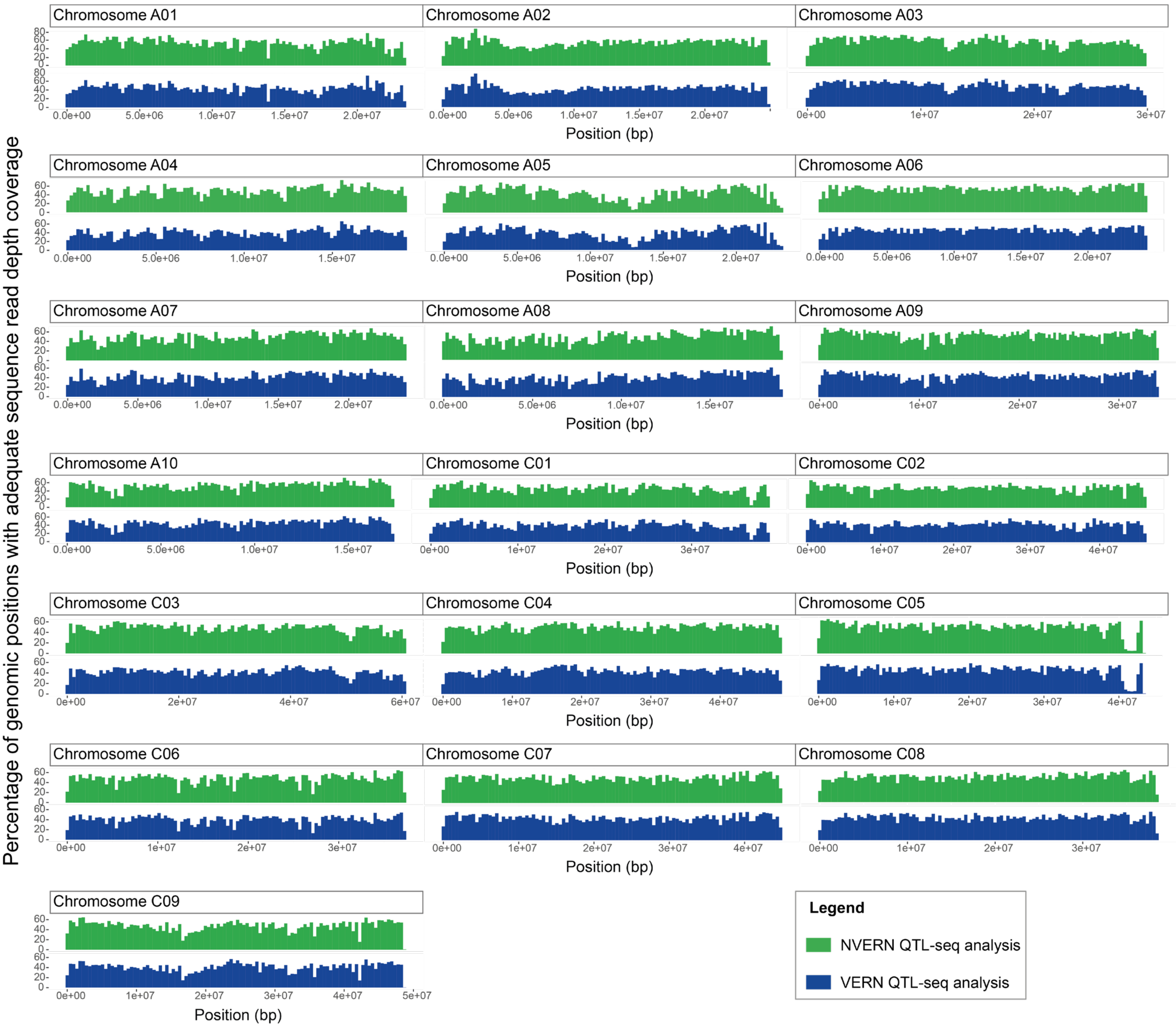
Percentage of genomic positions with adequate sequence read depth coverage
Chromosome A01
Chromosome A02
Chromosome A03
Chromosome A04
Chromosome A05
Chromosome A06
Chromosome A07
Chromosome A08
Chromosome A09
Chromosome A10
Chromosome C01
Chromosome C02
Chromosome C03
Chromosome C04
Chromosome C05
Chromosome C06
Chromosome C07
Chromosome C08
Chromosome C09
Position (bp)
Legend
NVERN QTL-seq analysis
VERN QTL-seq analysis
