# Supplementary material for: QTL‐seq identifies BnaFT.A02 and BnaFLC.A02 as candidates for variation in vernalization requirement and response in winter oilseed rape (Brassica napus)
Source: Plant Biotechnol J. 2020 Jun 23;18(12):2466–81. doi: 10.1111/pbi.13421 (PMC7680531; doi:10.1111/pbi.13421)
Supplement: Supplementary file 2 — Table S1 Summary of flowering time and Illumina sequencing data of parent lines and bulks for treatments VERN and NVERN. Table S2 KASP primers used for validation of QTL region. Table S3 Primers used to amplify and sequence BnaFLC.A02 and BnaFT.A02. Table S4 Primers used for quantitative RT‐PCR analysis of BnaFLC.A02 and BnaFT.A02. [file PBI-18-2466-s003.docx]

Table S1: Summary of flowering time and Illumina sequencing data of parental lines and bulks for treatments VERN and NVERN

| Sample | Number of bulked lines per sample | Mean days to flower after transfer to the poly-tunnel  (±95% confidence interval) | | Raw reads | Q30 (%) | GC (%) |
| --- | --- | --- | --- | --- | --- | --- |
|  |  | VERN | NVERN |  |  |  |
| Cabriolet | 1 | 42.92 ±0.89 | 78.00 ±1.53 | 121,756,072 | 92.34 | 37.42 |
| Darmor | 1 | 71.25 ±4.21 | 170.00 ±0.00 | 106,033,425 | 91.35 | 37.80 |
| VERN_EARLY | 37 | 42.35 ±0.25 |  | 106,467,543 | 91.19 | 37.97 |
| VERN_LATE | 32 | 69.75 ±1.70 |  | 110,166,633 | 90.26 | 37.89 |
| NVERN_EARLY | 34 |  | 75.26 ±0.57 | 129,129,964 | 91.93 | 37.31 |
| NVERN_LATE | 36 |  | 170.00 ±0.00 | 132,335,109 | 92.83 | 37.30 |

Table S2: KASP primers used for validation of QTL region

| Target | Name | SNP position | Direction | Fluorescence tail | KASP marker sequence |
| --- | --- | --- | --- | --- | --- |
| CONSERVED | C_A02_136553 | 136553 | R |  | TGCCTAGAGGGCTCCATACA |
| DARMOR | X_A02_136553 | 136553 | F | FAM | GAAGGTGACCAAGTTCATGCTCGAACTAATGGCTCATGGGTGC |
| CABRIOLET | Y_A02_136553 | 136553 | F | HEX | GAAGGTCGGAGTCAACGGATTCGAACTAATGGCTCATGAGTGG |
| CONSERVED | C_A02_1290192 | 1290192 | R |  | TGTCTGAGTGCCTCCTTGCA |
| DARMOR | X_A02_1290192 | 1290192 | F | FAM | GAAGGTGACCAAGTTCATGCTTCTGCTCTCTGAATTTTGAGATTA |
| CABRIOLET | Y_A02_1290192 | 1290192 | F | HEX | GAAGGTCGGAGTCAACGGATTTCTGCTCTCTGAATTTTGAGATTG |
| CONSERVED | C_A02_1997465 | 1997465 | R |  | AGCCAAGGATCCACATAATCA |
| DARMOR | X_A02_1997465 | 1997465 | F | FAM | GAAGGTGACCAAGTTCATGCTAGCAAGGAGAATGCATATTCTCA |
| CABRIOLET | Y_A02_1997465 | 1997465 | F | HEX | GAAGGTCGGAGTCAACGGATTAGCAAGGAGAATGCATATTCTCT |
| CONSERVED | C_A02_4654252 | 4654252 | F |  | TGCCTTGAGAAGGCTATGAGAG |
| DARMOR | X_A02_4654252 | 4654252 | R | FAM | GAAGGTGACCAAGTTCATGCTCCATAACTTGGTCTTTGGCAGC |
| CABRIOLET | Y_A02_4654252 | 4654252 | R | HEX | GAAGGTCGGAGTCAACGGATTCCATAACTTGGTCTTTGGCAGT |
| CONSERVED | C_A02_6375504 | 6375504 | R |  | CGTCTCCGACTTGTAACCCA |
| DARMOR | X_A02_6375504 | 6375504 | F | FAM | GAAGGTGACCAAGTTCATGCTGTTATGATCACCGATCCGAACT |
| CABRIOLET | Y_A02_6375504 | 6375504 | F | HEX | GAAGGTCGGAGTCAACGGATTTATGATCACCGATCCGAACC |

Table S3: Primers used to amplify and sequence *BnaFLC.A02* and *BnaFT.A02*

| Gene | Forward primer name | Forward primer sequence 5’ to 3’ | Reverse primer name | Reverse primer sequence 5’ to 3’ |
| --- | --- | --- | --- | --- |
| *BnaFLC.A02* | A02FLC_F | ACACGTGGCTGTCTTGTCCC | A02FLC_R1 | GGCTGCACAATGTGGCATATATG |
| *BnaFLC.A02* | A02FLC_F2 | TCTGGGTCTTCCTTTATTTGCCC | A02FLC_R2 | CCTCCAGCTGAACGAGGGAG |
| *BnaFLC.A02* | A02FLC_F3 | TGCCTATTCTATCCCTTCTCCG | A02FLC_R3 | CCTAACCAATCGGATCCCAA |
| *BnaFLC.A02* | A02FLC_F4 | GAATGAAGCCTGGATCTGGA | A02FLC_R4 | ACAGAAGAGACGAACACACACAG |
| *BnaFT.A02* | A02FT_F1 | GTGATGAGTTCACCGACCCG | A02FT_R1 | CGGAGGTGAGGATTGCTAGG |
| *BnaFT.A02* | A02FT_F3 | GCAGACYCATAAGGAGGCCTTA | A02FT_R3 | TGTTGCATGTGTGGTTCAGTG |
| *BnaFT.A02* | A02FT_F4 | CTTCTTCGTCTTATTTGTAACGG | A02FT_R4 | GTTTATCCTTGGTGTATGGGAC |

Table S4: Primers used for quantitative RT-PCR analysis of *BnaFLC.A02* and *BnaFT.A02*

| Gene | Forward primer name | Forward primer sequence 5’ to 3’ | Reverse primer name | Reverse primer sequence 5’ to 3’ | Reference |
| --- | --- | --- | --- | --- | --- |
| *BnaUBC* | BnUBC21_qF2 | GTCCTCTCAACTGCGACTCA | BnUBC21_qR2 | GTGTGTACATGCGTGCCATT | Orsel et al., 2014 |
| *BnaFLC.A02* | q41 | CCTCCGGCAAGCTTTACAAC | q42 | GAGCTTTTGACTGAAGATCCAGA | Hawkes 2017 |
| *BnaFT.A02* | BnC2FT_BnA2FT_Guo_Fw | GTTGTAGGAGACGTTCTTGAATGT | BnA2FT_Guo_Rev | TCTGGATCCACCATAACCAAAGTA | Guo et al., 2014 |
