# Supplementary material for: QTL‐seq identifies BnaFT.A02 and BnaFLC.A02 as candidates for variation in vernalization requirement and response in winter oilseed rape (Brassica napus)
Source: Plant Biotechnol J. 2020 Jun 23;18(12):2466–81. doi: 10.1111/pbi.13421 (PMC7680531; doi:10.1111/pbi.13421)
Supplement: Supplementary file 3 — Supplementary Material [file PBI-18-2466-s001.docx]

**Supplementary legends:**

**Figure S1:** Temperature and humidity recorded within the Keder plastic poly-tunnel during the 2017 flowering time phenotyping analysis of F_2_ lines at JIC.

**Figure S2:** Frequency distributions of sequence variants (SNPs and small InDels) detected in Darmor compared with Darmor-*bzh*. Each chromosome is plotted separately, and the frequency of variants detected are plotted by genome order.

**Figure S3:** Frequency distributions of sequence variants (SNPs and small InDels) detected in Cabriolet compared with Darmor. Each chromosome is plotted separately, and the frequency of variants detected are plotted by genome order.

**Figure S4:** No sequence variants (SNPs and small InDels) are detected in Cabriolet compared with Darmor at the major flowering time genes *BnaFRI.A03* and *BnaFLC.A10*. (A) The relative position of *BnaFRI.A03* on chromosome A03 is plotted with the closest sequence variant found up- and down-stream highlighted with a red arrow. (B) The relative position of *BnaFLC.A10* on chromosome A10 is plotted with the closest sequence variant found up- and down-stream highlighted with a red arrow.

**Figure S5:** Visualization of homeologous genome exchanges in Cabriolet and Darmor based on DNA resequencing.

(A & B): The relative redundancy of coverage of A and C genome homeologous gene pairs is represented in CMYK colour space, with cyan component representing coverage of the *Brassica* A genome copy and magenta component representing coverage of the *Brassica* C genome copy.

1. Genome-wide homeologous genome exchanges in Cabriolet and Darmor. The gene pairs are plotted in Brassica C genome order (chromosomes denoted C1 to C9)
2. Homeologous exchanges present on chromosome A02/C02. The gene pairs are plotted in Brassica chromosome C02 gene order, the relative position of *BnaFLC.A02/C02* and *BnaFT.A02/C02* gene pairs are highlighted

**Figure S6:** Expression of *BnaFLC.A02* varies between Cabriolet and Darmor before and after vernalisation.

(A-D) Normalised expression of *BnaFLC.A02* in Cabriolet, Darmor, and genotyped F_2_ individuals as measured by quantitative RT-PCR before (NV) and after vernalisation (6WT0, 6WT16, 6WT30). The expression levels, normalised to *UBC21*, detected in each line plant are plotted here.

1. Normalised expression of *BnaFLC.A02* detected in 3 Cabriolet, 3 Darmor and 10 F_2_ individuals with genotypic combination BnaFLC.A02-Dar/BnaFT.A02-Dar
2. Normalised expression of *BnaFLC.A02* detected in 3 Cabriolet, 3 Darmor and 10 F_2_ individuals with genotypic combination BnaFLC.A02-Dar/BnaFT.A02-Cab
3. Normalised expression of *BnaFLC.A02* detected in 3 Cabriolet, 3 Darmor and 10 F_2_ individuals with genotypic combination BnaFLC.A02-Cab/BnaFT.A02-Dar
4. Normalised expression of *BnaFLC.A02* detected in 3 Cabriolet, 3 Darmor and 10 F_2_ individuals with genotypic combination BnaFLC.A02-Cab/BnaFT.A02-Cab

**Figure S7:** Expression of *BnaFT* is detected in Cabriolet, but not detectable in Darmor

1. Normalised expression of *BnaFT.A02* in Cabriolet and Darmor over time and under ambient temperature conditions as measured by quantitative RT-PCR error bars denote one standard error around the mean calculated from at least three biological replicates, T= days from sowing
2. Normalised expression of *BnaFT.A02* in Cabriolet and Darmor as measured by quantitative RT-PCR before (NV) and after vernalisation (6WT0, 6WT16, 6WT30), error bars denote one standard error around the mean calculated from three biological replicates
3. Normalised expression of *BnaFT.C02* in Cabriolet and Darmor as measured by quantitative RT-PCR before (NV) and after vernalisation (6WT0, 6WT16, 6WT30), error bars denote one standard error around the mean calculated from three biological replicates
4. Normalised expression of *BnaFT.A02* in F_2_ lines genotyped for *BnaFLC.A02* and *BnaFT.A02* as measured by quantitative RT-PCR before (NV) and after vernalisation (6WT0, 6WT16, 6WT30), error bars denote one standard error around the mean calculated from at least three biological replicates
5. Normalised expression of *BnaFT.A02* in F_2_ lines genotyped for *BnaFLC.A02* and *BnaFT.A02* as measured by quantitative RT-PCR before (NV) and after vernalisation (6WT0, 6WT16, 6WT30), error bars denote one standard error around the mean calculated from at least three biological replicates

**Figure S8:** The distribution of genomic positions included in the QTL-seq analysis. Each chromosome is plotted as separate histograms of the frequency of genomic positions with read depth coverage of more than 20 reads and included in SNP and ΔSNP indices calculation.
